# Traces of Late Bronze and Early Iron Age Mongolian Horse Mitochondrial Lineages in Modern Populations

**DOI:** 10.3390/genes12030412

**Published:** 2021-03-12

**Authors:** Mariya A. Kusliy, Nadezhda V. Vorobieva, Alexey A. Tishkin, Alexey I. Makunin, Anna S. Druzhkova, Vladimir A. Trifonov, Tumur-O. Iderkhangai, Alexander S. Graphodatsky

**Affiliations:** 1 Department of the Diversity and Evolution of Genomes, Institute of Molecular and Cellular Biology SB RAS, 630090 Novosibirsk, Russia; vorn@mcb.nsc.ru (N.V.V.); alex.makunin@gmail.com (A.I.M.); rada@mcb.nsc.ru (A.S.D.); vlad@mcb.nsc.ru (V.A.T.); graf@mcb.nsc.ru (A.S.G.); 2 Department of Archaeology, Ethnography and Museology, Altai State University, 656049 Barnaul, Russia; tishkin210@mail.ru; 3 Department of Archaeology, Ulaanbaatar State University, Ulaanbaatar 13343, Mongolia; iderkhangai83@gmail.com

**Keywords:** ancient DNA, mitochondrial DNA, Mongolian horse, phylogeography

## Abstract

The Mongolian horse is one of the most ancient and relatively unmanaged horse breeds. The population history of the Mongolian horse remains poorly understood due to a lack of information on ancient and modern DNA. Here, we report nearly complete mitochondrial genome data obtained from five ancient Mongolian horse samples of the Khereksur and Deer Stone culture (late 2nd to 1st third of the 1st millennium BC) and one ancient horse specimen from the Xiongnu culture (1st century BC to 1st century AD) using target enrichment and high-throughput sequencing methods. Phylogenetic analysis involving ancient, historical, and modern mitogenomes of horses from Mongolia and other regions showed the presence of three mitochondrial haplogroups in the ancient Mongolian horse populations studied here and similar haplotype composition of ancient and modern horse populations of Mongolia. Our results revealed genetic continuity between the Mongolian horse populations of the Khereksur and Deer Stone culture and those of the Xiongnu culture owing to the presence of related mitotypes. Besides, we report close phylogenetic relationships between haplotypes of the Khereksur and Deer Stone horses and the horses of indigenous breeds of the Middle East (Caspian and Iranian), China (Naqu, Yunnan, and Jinjiang), and Italy (Giara) as well as genetic similarity between the Xiongnu Mongolian horses and those of the most ancient breeds of the Middle East (Arabian) and Central Asia (Akhal-Teke). Despite all the migrations of the Mongolian peoples over the past 3000 years, mitochondrial haplogroup composition of Mongolian horse populations remains almost unchanged.

## 1. Introduction

The Mongolian horse breed is referred to as a “landrace breed” because these horses are free-ranging and to a lesser extent have experienced selective pressures applied by breeders [1]. Mongolian horse populations are believed to have been the source for many breeds across Asia [2], including Tuva [1], Yunnan [3], and Jeju [4,5,6]. The assumption that the ancient Mongolian horse population might have been ancestral to many modern horse breeds [3] is also supported by the highest levels of within-breed diversity according to genome-wide autosomal, microsatellite, protein, and mitochondrial polymorphism data [1,3,7,8]. Even though the Mongolian horse is one of the most ancient breeds and is considered ancestral for many other breeds, very little research on its ancient DNA has been carried out, though abundant osteological material has been collected [9,10,11]. Only two mitogenomes of historical Mongolian horses are available in GenBank, and even these mitogenomes are not older than the 20th century. A few ancient horse populations of Mongolia have been investigated at the mitochondrial level [4,12,13]. These studies have determined hypervariable region mitotypes of three ancient horses found in burials of the Early Iron Age Pazyryk archaeological sites (Olon-Kurin-Gol-6 and Olon-Kurin-Gol-10) in Northwestern Mongolia [13]. According to the classification of Cieslak et al. [12], the three horses carry the haplotypes that were widespread in the Iron Age in China, Southwest Siberia, and Kazakhstan [13]. A comparison of mitochondrial control region diversity between ancient and modern Mongolian horse populations has revealed that the Pazyryk horse haplotypes also occur in modern populations. However, modern populations also contain other haplotypes. It has been shown that many East Asian mitochondrial lineages of Bronze and Iron Age were missing in other regions of the Eurasian steppe (West Siberia, South Siberia, Kazakhstan), possibly owing to the isolation of Mongolian and Chinese wild horses by the Altai Mountains and the Takla Makan and Gobi deserts [12]. To date, only one study has been conducted in which complete mitochondrial genomes were obtained for the Mongolian ancient horses of the Khereksur and Deer Stone culture and the Xiongnu culture. Phylogeographic reconstructions in this research showed that Mongolian horses of the Bronze Age Khereksur and Deer Stone culture were related to the Bronze Age horses of Moldova and Germany and 5500-year-old Botai culture horses of the Central Asian steppes. The results of that study also revealed that the horses of the Iron Age Xiongnu culture were closely related to the horses of the Neolithic period from the Orenburg region of Russia and those of the Gallo-Roman period of classical antiquity from France [4].

We carried out a molecular genetic study of bone samples of ancient horses associated with the Ganga Tsagaan ereg (late 12th to mid-10th century BC) and the Ereen hailaas (1st century BC to 1st century AD) archaeological sites, belonging to the Khereksur and Deer Stone archaeological culture and the Xiongnu archaeological culture, respectively. The above-mentioned archaeological sites are located close to each other in the valley of the Egiin gol river in the territory of Northern Mongolia. The Ganga Tsagaan ereg archaeological site belongs to the Khereksur and Deer Stone culture, which represents the most archaic nomadic empire [14]. No settlement of this culture has been found yet [14,15]. Most of the investigated kurgans (khereksurs) are characterized by the presence of so-called altars (small stone mounds), under which a horse skull as well as some cervical vertebrae bones and hooves have been found. This ritual (symbolic) action implied an accompanying burial of a horse for the afterlife. No deer stones have been found at the Ganga Tsagaan ereg site, thus indirectly indicating an earlier time point of the khereksur construction in comparison with those with deer stones [15]. Archaeological and paleozoological data indicate that clear evidence of widespread use of domesticated horses in Mongolia dates back to the end of the 2nd millennium BC and is associated with the Khereksur and Deer Stone culture [16,17]; ancient people of this culture mainly bred horses, sheep, and cattle [16]. The Ereen hailaas archaeological site (Xiongnu culture) is related to the period when the location of the Shanyu (leader of the Xiongnu Empire) was moved from the Chinese Han Empire far to the north, and the Huns began to explore the territory of modern Mongolia as well as Buryatia (Russia) and Transbaikalia (Russia). The Xiongnu society was characterized by an economy based on nomadic animal husbandry. The nomads of the Xiongnu Empire bred animals traditional for the Eurasian steppes: horses, sheep, goats, camels, and cattle. Of all these species, horses had the highest economic and military significance and had a special place in the culture of ancient nomads [18]. The results of paleofaunistic studies have shown that by their exterior features, most of the Xiongnu horses were similar to the horses of the Mongolian type [19]. However, it cannot be ruled out that some of the horses of the elite were Central Asian horses (Akhal-Teke horse) because similar horses are depicted on burial mound drapery from the Noin-Ula site (Xiongnu culture) [20].

The aim of this study was to determine mitochondrial diversity of ancient Mongolian horse populations of the Khereksur and Deer Stone (Ganga Tsagaan ereg site) archaeological culture and the Xiongnu (Ereen hailaas site) archaeological culture, their continuity, and phylogeographic relationships by means of mitogenome target-enriched high-throughput sequencing data. As a result, we report nearly complete mitogenomes from six ancient Mongolian horses, phylogeographic analysis of which, together with previously published horse mitogenomes, revealed the presence of similar mitotypes in the studied ancient Mongolian populations and close phylogenetic relationships between the horses studied here and modern horses of the most ancient and indigenous breeds of Central and Eastern Asia, the Middle East, and the Mediterranean region.

## 2. Materials and Methods

### 2.1. Information about the Samples

Five osteological specimens of ancient horses from Mongolian archaeological site Ganga Tsagaan ereg (Khereksur and Deer Stone culture, late 12th to mid-10th century BC) and one tooth specimen of an ancient horse from Mongolian archaeological site Ereen hailaas (Xiongnu culture, 1st century BC to 1st century AD) were investigated in this work (detailed information about the samples is given in Table S1).

### 2.2. Ancient-Mitogenome Sequencing

All experiments were conducted at the Institute of Molecular and Cellular Biology ofthe Siberian Branch of the Russian Academy of Sciences (SB RAS) (Novosibirsk, Russia), in a special laboratory focused only on the research on ancient DNA, with maximum protection against contamination (use of special protective clothing, surface treatment with DNAZap nucleic-acid–degrading solutions, and ultraviolet irradiation of laboratory premises). All ancient bone specimens were subjected to UV irradiation (30 min on each side of a bone); 1 cm^2^ of bone (0.3 g) was sawed off with a diamond disk of an electric drill, the surface layer was removed, and the bone was crushed into a powder in a metal mortar. Ancient-DNA extraction was performed following the protocol described in the article of Yang et al. [21] with one modification made by Sanderson, Radley, Mayton [22]: lysis buffer contained NH_4_-EDTA for reducing decalcification time from 18–24 to 1.5–3.0 h at a standard concentration of 300 mg of the bone powder per 5 mL of the buffer at 55 °C. Genomic libraries of ancient DNA fragments were obtained using the TruSeq Nano DNA Sample Preparation Kit (Illumina, San Diego, CA, USA) according to the manufacturer’s protocol: High Sample Protocol with a few modifications: samples were purified with the MinElute PCR Purification Kit (Qiagen, Hilden, Germany) instead of purification on the AMPure XP beads (Illumina), and 12 cycles of library amplification were performed.

Two rounds of library enrichment were carried out by hybridization with biotinylated modern *Equus caballus* mitochondrial DNA (mtDNA) immobilized on DynabeadsVR Streptavidin magnetic particles (Life Technologies), according to a previously published method [23] with modifications, described in the article of Vorobieva et al. [24]. Amplification of the enriched libraries was carried out in a volume of 50 μL containing 1× Phusion HF Buffer, 0.2 mM each dNTP, 1 μM each primer for library fragment adapters (SuD Nano DNA Library Prep Kit for Illumina), 1 unit of Phusion DNA polymerase. Cycling conditions included 30 s initial denaturation at 95 °C; followed by 20 cycles of 20 s denaturation at 98 °C, 20 s primer annealing at 65 °C, and 20 s elongation at 72 °C; with final 5 min elongation at 72 °C.

The obtained libraries were quantified by real-time PCR in the presence of dye SYBR Green 1 and reference dye for quantitative PCR (ROX), using a PCR kit: 2.5× reaction mix (Syntol), according to the manufacturer’s protocol. Contamination was monitored through extraction, library, and amplification blanks, which all yielded negative results. Paired-end sequencing of the enriched libraries was performed on the MiSeq platform (Illumina) with the MiSeq v2 Reagent Kit (300 cycles, 2 × 150 bp).

### 2.3. Sequence Data Analysis

For the initial sequencing data processing, PALEOMIX BAM Pipeline v1.3.2 was used [25]. Reads were trimmed and collapsed (AdapterRemoval v.2.2.2) [26]. Sequencing read alignments against reference sequences of the horse mitogenome (GenBank accession No.: NC_001640.1) and the human mitogenome for contamination control (GenBank accession No.: NC_012920.1) were performed using sequence aligner bwa v0.7.15 [27] with the following parameters: algorithm, mem; minimum mapping quality, 25; and the FilterUnmappedReads option. The alignment improvement included filtering of PCR duplicates (paleomix rmdup_collapsed). To obtain more reliable sequences of the mitochondrial genomes, we performed rescaling of the base quality scores in the BAM alignment file, according to the base’s probability of being affected by postmortem damage, and constructed a postmortem DNA damage model using the MapDamage v2.2.0 computational framework [28]. To remove human DNA contamination, a custom script (https://github.com/lca-imcb/lca-ngs/blob/master/contam_filter.py (accessed on 19 February 2021)) based on mapping quality comparison was employed. Consensus sequences were called using a minimum depth of coverage of 2 and filtering for base quality ≥30; all variants alternative to the reference sequence were confirmed on bioinformatics software platform Geneious Prime v2020.2.4 (https://www.geneious.com (accessed on 19 February 2021)) with a set threshold of the highest quality (bases had to match at least 60% of total adjusted quality). The depth and width of the mitochondrial genome coverage were determined on software platform Geneious Prime v2020.2.4.

### 2.4. Phylogenetic Analysis

Multiple alignment of the mitogenome consensus sequences was conducted using multiple sequence alignment program MAFFT v7.407 [29]. To select best-fit partitioning schemes and models of molecular evolution for phylogenetic analyses, we utilized PartitionFinder v2.1.1 [30]. In the horse mitochondrial genome alignment, we identified 5 partitions with the following evolutionary models of nucleotide substitutions: HKY + I for the second codons of protein-coding genes; HKY + G + I for RNA-coding genes, first and third codons of protein-coding genes; and GTR + G + I for hypervariable regions. A time-measured Bayesian phylogenetic tree was constructed including 206 horse mitogenomes via the above-mentioned partitioning schemes and evolutionary models on an advanced software platform for Bayesian evolutionary analysis, BEAST v1.10.4 [31] (50 million generations of the Markov Chain, sampling frequency: 1000, the first 10% of the trees were discarded as burn-in), clock model: strict clock [32], tree prior: coalescent (constant population size) [33,34], and the tree model: random starting tree. The branch divergence times were derived using the ages of all ancient samples as internal calibrations at the tips of the evolutionary tree. All specimens used for the phylogenetic reconstructions were dated either in calibrated years before the present or years before the present associated with the corresponding archaeological context (Table S2). The BEAST configuration file was generated in BEAUti v1.10.4, and the BEAST output was analyzed in Tracer v1.7.1 [35]. In the latter, effective sample sizes for all the traces were greater than 800, and posterior probability density was bell-shaped, suggesting that the parameter estimates reached convergence. To summarize the information contained within our sampled trees, we used a tool called TreeAnnotator v1.10.4 [36]. The annotated tree was visualized using the FigTree v1.4.4 software (http://tree.bio.ed.ac.uk/software/figtree/ (accessed on 22 February 2021)).

### 2.5. Data Availability

All studied bone and tooth samples (Er1, Gan1, Gan3, Gan11, Gan14, and Gan18) of ancient horses are stored in a repository located in the Department of Archaeology of Ulaanbaatar State University, Ulaanbaatar, Mongolia. The samples were provided by a coauthor of this article, Dr. Tumur-Ochir Iderkhangai. The present study is compliant with all relevant laws and regulations. All the experiments were approved by the Ethics Committee on Animal and Human Research at the Institute of Molecular and Cellular Biology, Russia (permit No. 01/20 of 11 February 2020).

The next-generation sequencing data were submitted to the Sequence Read Archive database (SRA) and are available under BioProject accession number PRJNA694234 (https://www.ncbi.nlm.nih.gov/bioproject/?term=PRJNA694234 (accessed on 23 February 2021)). GenBank accession numbers of the mitogenome consensus sequences are as follows: MW534078 for sample Er1, MW534079 for sample Gan1, MW534080 for sample Gan3, MW534081 for sample Gan11, MW534082 for sample Gan14, and MW534083 for sample Gan18.

## 3. Results

### 3.1. Authenticity of Ancient DNA Data

As already determined, the main characteristics of ancient DNA molecules are cytosine deamination resulting in an increased C → T substitution rate and an increased complementary G → A substitution rate towards 5″ and 3″ read termini, respectively, as well as DNA degradation to small fragments, generally below 100 bp [37]. Using the MapDamage v2.2.0 computational framework, postmortem damage and fragmentation patterns in the obtained ancient DNA libraries were evaluated (Figures S1 and S2, respectively). All the ancient samples studied here showed expected nucleotide misincorporation signatures of postmortem DNA damage, proving the authenticity of the ancient DNA. However, the 5″ C-to-T damage profile was less pronounced due to peculiarities of the library preparation procedure (related to the blunt-end repair process, when the polymerase removes the 3″ overhangs and fills in the 5″ overhangs of the fragments). The average size of DNA fragments of the studied libraries varied from 60 to 90 bp, which also points to the antiquity of the target DNA. Among the studied samples, the youngest sample of the Xiongnu culture (Er1) has the largest average length of DNA fragments, indicating that in our sample set, the degree of DNA degradation depends on sample age. However, the conditions of sample preservation were slightly different. The bone remains of the Er1 horse were located in a pit along with the osteological material of other animals, while the bone specimens of each Ganga Tsagaan ereg horse were buried in a separate altar and were well isolated from other structures of the ritual complex [15]. We believe that the lower genome coverage of the Er1 sample results from a higher level of exogenous DNA that can be attributed to the difference in sample location (the pit rather than an altar for other samples). The amount of human contamination removed using the custom script (https://github.com/lca-imcb/lca-ngs/blob/master/contam_filter.py (accessed on 23 February 2021)) ranged from 0 to 150 reads per sequencing library.

### 3.2. Characteristics of the Ancient Mongolian Horse Mitochondrial Genomes

The width of coverage of the obtained mitochondrial genomes of the ancient horses varies from 97.3% to 99.8% of the reference sequence length, and the average depth of coverage is between 6.7-fold and 85.9-fold. The ratio of the unique mapped collapsed reads to the total number of collapsed reads is 1.2% on average. We used the obtained sequences of the nearly complete mitochondrial genomes of the ancient Mongolian horses in our phylogenetic reconstructions. Detailed main characteristics of the obtained mitogenomes are shown in Table 1. Coverage depth of the obtained mitogenomes of ancient Mongolian horses is visualized in Figure S3.

### 3.3. Phylogenetic Reconstructions

**Figure 1:**
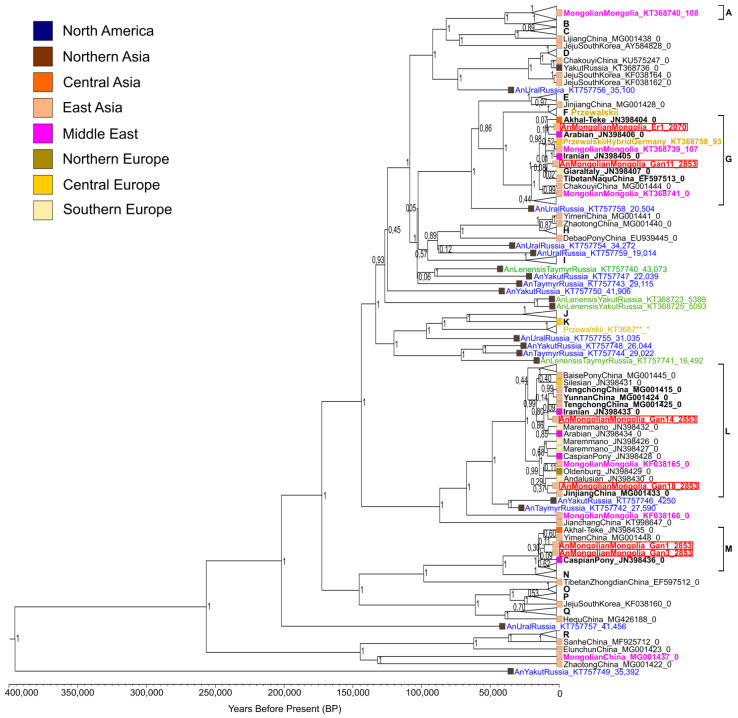
The Bayesian phylogenetic tree of the ancient Mongolian horses studied here and ancient and modern horses of different origins. The tree was constructed based on the alignment of 6 sequences of mitogenomes obtained here and 200 previously published ones (Table S2). Mitochondrial haplogroups A–R by classification of Achilli et al. [38] are marked in black. The red color of a sample name denotes the ancient horses of Mongolia studied here, the green color indicates those belonging to the *Equus lenensis* lineage, golden means belonging to Przewalski’s horse, blue denotes ancient horses outside Mongolia, pink means modern and historical (20th century) horses of Mongolia, and black indicates modern horses outside Mongolia. Sample names consist of three parts separated by underscores. The first part is a species affiliation or horse breed name and geographic origin of the sample, the second part is a GenBank accession number, and the third part is sample age. The color palette of the squares indicates the geographical origin of samples. The tree was rooted by means of a published donkey (*Equus asinus*) mitochondrial genome (GenBank accession No.: NC_001788.1) (not displayed). Numbers near the tree branch nodes indicate posterior probability of the topology obtained by the Bayesian method. The time divergence scale at the bottom of the figure is a timeline where the dates are expressed in years before the present. Tree nodes with modern horses, not considered in the Discussion section, were collapsed for better visualization.

By the Bayesian method, a phylogenetic tree was constructed (Figure 1) based on the obtained mitogenome sequences and the mitogenome sequences of modern horses of different breeds investigated in the study by Achilli et al. [38], in which there have been identified 18 major mitochondrial haplogroups (A–R) and 83 mitotypes. Due to a lack of mitogenome sequences from ancient and modern horses of Asian breeds, this set of sequences was supplemented with mitogenomes from the GenBank sequence database, with special attention paid to the horse breeds that are considered closely related to the Mongolian horse (Tuva, Yakutian, Yunnan, and Jeju [1,3,4,6]) and those with origins in East Asia. Information on the mitogenome sequences used to build the phylogenetic tree is given in Table S2. Initial trees that display the 95% confidence intervals around the mean divergence time are presented in Figures S4 and S5 (with and without *Equus asinus* (outgroup), respectively).

Almost all ancient horses outside Mongolia shown in the phylogenetic tree must have been wild (*Equus ferus* lineage). They are located in the tree branches not belonging to any domesticated horse haplogroups identified by Achilli et al. [38] and therefore are most likely to be extinct clades of evolution. All these extinct lineages sampled so far originated from the territories of the Ural Mountains and Western and Eastern Siberia (Russia) [39,40]. According to the literature data, horses of the extinct *Equus lenensis* lineage inhabited the territories of Eastern and Western Siberia from the Late Pleistocene to the Middle Holocene [4,41]. The historical and modern Przewalski’s horses (*Equus ferus przewalskii*) and their hybrids with domestic horses (*Equus ferus caballus*) are also not related to the ancient Mongolian horses analyzed here. None of the studied ancient horses of Mongolia are located in the same clade with Lena horses (*Equus lenensis*) in the phylogenetic tree. Consequently, the mitotypes of the ancient Mongolian horses are within mitochondrial diversity of domestic horses.

Ancient horses from the Ganga Tsagaan ereg archaeological site (Khereksur and Deer Stone culture) were found to belong to mitogroups G, L, and M identified by Achilli et al. [38] and the ancient horse from the Ereen hailaas site (Xiongnu culture) was shown to belong to mitogroup G. Consequently, this haplogroup was present in the ancient horse populations of Mongolia, both in the Khereksur and Deer Stone culture and in the Xiongnu culture, thus indicating certain continuity between the horse populations of these Mongolian archaeological cultures. Comparing the obtained results with those of a recently published study [24], we can conclude that the studied ancient Mongolian horse populations of the Late Bronze Age and the Early Iron Age were less diverse in mitochondrial composition than the Ukok Altai horses of the Early Iron Age. Six Ukok horses have been assigned to five different mitogroups: A, I, N, P, and Q, as classified by Achilli et al. [38].

According to the published data, haplogroup G is more common in Asia and has lower prevalence in the Middle East and Southern Europe. Haplogroup L is more common in Europe, becoming less and less common toward the East, albeit containing many mitotypes of different Chinese breeds. Haplogroup M has the same prevalence in Europe and Asia. Haplogroups G and M were more common among ancient horse populations as compared to modern ones [38]. Therefore, it is not surprising that the four ancient horses of Mongolia under study fell into these haplogroups.

We noticed a connection between the khereksur number and the mitogroup of horses whose bone remains were found in the altars, next to the khereksurs. Horses of the Ganga Tsagaan ereg site associated with the same khereksur belong to the same mitochondrial haplogroup. Horses Gan14 and Gan18 belonging to mitogroup L were buried in altars number 10 and number 14, respectively, next to khereksur 1-082. Horses Gan1 and Gan3 belong to the same haplogroup M, and their bone remains were found in altars number 2 and 4, respectively, next to khereksur 1-078 [15]. We inferred that most likely these studied ancient horses of different haplogroups belonged to different people and were bred independently of each other.

### 3.4. Time of Haplotype Divergence

On the basis of the constructed Bayesian phylogenetic tree (Figure 1), we estimated the time of divergence of the identified mitotypes and the closely related ones. Age estimates of the relevant nodes with 95% HPD (height posterior density) are shown in Table 2. Most Mongolian ancient horse mitotypes and the closest genetically related mitotypes diverged ~5000 years ago (median age estimate). Only the ancestral mitotype of two ancient Mongolian horses of the Khereksur and Deer Stone culture and the (closest to them) modern horse of the Caspian pony breed existed ~10,000 years ago (median age estimate). The extinct genetic lineages of wild horses from Eastern and Western Siberia located in the basal positions in relation to the main haplogroups and the lineages of domesticated horses diverged within the last 50–390 thousand years (median age estimates). Domesticated horses of haplogroup L and wild horses from Yakutia (the youngest sample of a wild horse: 4300 years [39]) and from the Taymyr Peninsula have the latest divergence time (median age estimate: 50,000 years). We can conclude that the divergence times of mitotypes of the studied Mongolian ancient horses and the closest related modern horse mitotypes belong the time span from the Upper Paleolithic to the Early Iron Age (95% HPD: [2330.9; 14,467.8]).

## 4. Discussion

### 4.1. Phylogeographic Relationships

**Figure 2:**
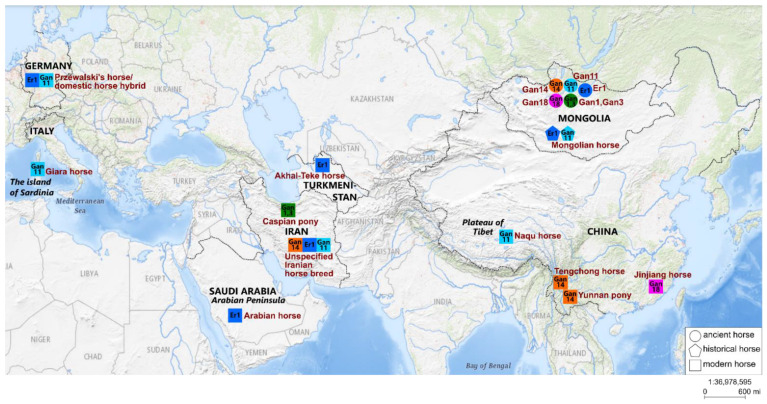
Geographical origin of the mitotypes closely related to the ancient ones obtained here. Circles represent ancient horses, and squares mean modern horses. Identical colors of circles and squares indicate similar mitotypes. Brown names are the names of ancient horse specimens and horse breeds. Black names and boundaries denote geographical objects within which the origin regions of closely related mitotypes are located. The figure was prepared based on the map in the USGS (United States Geological Survey) National Map Viewer (public domain) resource; it is similar but not identical to the original image and is therefore for illustrative purposes only.

The geographic origin data on the horse mitotypes closely related to the ancient Mongolian ones obtained here are depicted in Figure 2.

After detailed consideration of the phylogenetic relationships within haplogroup G, it becomes clear that ancient Mongolian horse Er1 of the Xiongnu culture falls into the same clade with modern horses of the Arabian and the Akhal-Teke Middle Eastern breeds. The Arabian horse breed is one of the oldest in the world. A recent study involving equine single-nucleotide polymorphism (SNP) arrays and whole-genome resequencing uncovered multiple origins of Arabian horses from the Middle East region [42]. The Akhal-Teke horse breed is also one of the most ancient. This is a breed of oriental origin, indigenous to Central Asia in the area of Turkmenistan from the Caspian coast to the Fergana Valley. According to historical data, this breed was developed several thousand years ago [43]. Another study, by means of historical, faunal, genetic, and iconographic data, has shown that the breed ancestral to Akhal-Teke horses is the horses ridden by the Pazyryk chieftains (4th to 2nd century BC). It has been suggested that Akhal-Teke horses were obtained by crossing the domesticated horse from the Middle Volga with the tarpan (*Equus ferus ferus*) of the Eurasian steppes [44]. From this information, we can assume that the ancient Mongolian horse of the Xiongnu culture studied here is closely related to modern horses of the most ancient breeds with origins in the Middle East and Central Asia.

One of the ancient horses, Gan11 from the Ganga Tsagaan ereg archaeological site of the Khereksur and Deer Stone culture, is located in the same clade with modern horses of the Tibetan Naqu breed and the Italian Giara breed. The Naqu horse is a small indigenous breed of China, it includes Tibetan-type horses with strong adaptation to the harsh environment of the Tibet region and areas adjacent to the Qinghai–Tibet Plateau [45]. The genetic kinship between the Tibetan and the Mongolian horse breeds has also been proven in a study based on the analysis of microsatellite markers [46]. The Giara horse is one of the 15 extant native Italian breeds and is geographically isolated on the island of Sardinia [47]. Based on phylogenetic reconstruction, it can be concluded that the ancient Mongolian horse Gan11 is closely related to the modern horses of the native breeds of the Qinghai-Tibet Plateau in East Asia and the island of Sardinia in the Mediterranean region; this finding is quite interesting because these regions are geographically very distant.

Given that the clades with ancient horses Er-1 and Gan-11 are not well supported, we would like to consider their sister clade, which includes the modern Mongolian horse, a hybrid of Przewalski’s horse, a modern horse, and a horse of an unspecified Iranian breed. The similarity of the mitotypes of modern and ancient Mongolian horses from archaeological cultures of different periods indicates that some ancient mitotypes were present in Mongolian horse populations for several thousand years. The geographic origins of the Iranian horse and of the aforementioned Arabian horse are the same: they originated in the Middle East region. Taking into account all the above information, we can infer that the clade with ancient Mongolian horses Er-1 and Gan-11 mainly includes horses originating in Asia.

The haplotype of ancient horse Gan14 from the Ganga Tsagaan ereg archaeological site belongs to haplogroup L and forms a clade with modern horses of the Middle East (an unspecified Iranian breed) and Chinese horse breeds. This clade contains horses of the Chinese Yunnan pony and Tengchong breeds, which belong to the southwest Chinese group of horse breeds: the Yunnan Sichuan horses. Horses of this group are small, short, and slender and originate from the mountainous areas of southwest China; phenotypically, they are very different from the Tibetan horse and Northern China horse (including the Mongolian horse) [45,48,49]. Another study, involving an analysis of protein polymorphisms, has revealed that the Mongolian horse is the breed ancestral to the Yunnan horse [3]. The analysis of the mtDNA control region sequences has also confirmed the hypothesis that domestic horses of China descended from both imported (outside of China) and local horses, whereas the imported horses were introduced from the northern regions of the Eurasian steppe [50].

The ancient horse Gan18 studied here is genetically closest to the modern horse of Chinese breed Jinjiang (both belong to haplogroup L). The Jinjiang horse is a unique modern breed distributed in the coastal areas of Central and South East China and is most genetically and phenotypically distinct from other southern horse breeds of China [48,49,51]. Jinjiang horses have muscular bodies and the draft-type conformation and exhibit specific adaptations to the hot and humid conditions of the south coastal areas, whereas other southern horse breeds are usually slim and dwarflike. Despite these phenotypic differences, the Yunnan and Jinjiang horses are genetically closely related, as proven by analyzing the whole-genome SNPs [51]. 

From the above information, it can be concluded that the ancient horse Gan14 studied here shares ancestry with modern horses of the Iranian and the Yunnan horse breeds, which are phenotypically different from the Tibetan and the Mongolian horses. Horse Gan18 is most closely related to the Jinjiang horse, which phenotypically differs from Yunnan horses, but they are closely related to each other. Taking into account the above consideration, it can be supposed that the mitotypes of Chinese horses related to the ancient Mongolian ones originate from Mongolia.

Two ancient horses, Gan1 and Gan3 from the Ganga Tsagaan ereg archaeological site, belong to mitogroup M and are especially close to one of its mitotypes comprising the modern horse of the Caspian pony breed. The Caspian pony is one of the oldest breeds with origins in the Middle East and was most likely developed on the territory of northern Iran during the Achaemenid Empire ~2.5–3.0 thousand years ago. Caspian ponies are horses of small stature and have probably resulted from natural hybridization between *Equus ferus caballus* and *Equus ferus przewalskii* [52]. However, this hypothesis has not yet been proven. Based on the analysis of a genome-wide set of autosomal SNPs, it has been determined that the Caspian horse falls into a clade with the other Middle Eastern breeds, the Arabian and the Akhal-Teke [1]. It can be assumed that the ancient horses of the Ganga Tsagaan ereg site are closely related to some Caspian horses of Middle Eastern origin.

### 4.2. Cultural Context

The Mongolian horse is one of the most ancient breeds, is relatively unmanaged, and has existed on the same territory for thousands of years [1], and the mitotypes closely related to the ancient Mongolian ones belong to modern horses of the breeds that—according to the literature data—are quite ancient (several thousand years) and indigenous [42,43,45,47,48,49,51,52]. Therefore, one can suggest possible migration routes of the obtained ancient mitotypes. However, due to the limited dataset, further research on ancient DNA of horses belonging to earlier archaeological cultures is needed for more accurate determination of these migration pathways.

The five studied ancient horses (Ganga Tsagaan ereg site) belong to the Khereksur and Deer Stone nomadic culture of Mongolia. The use of horses and camels provided mobility and some military superiority for the nomads over the farmers of Eurasia in the preindustrial era [53]. The widespread use of ritual horse sacrifices, typical of this culture, occurred at the same time as the spread of horse riding, horse milk utilization, and a significant increase in the proportion of horse meat in the human diet in most of Central Asia [54]. The Mongolian tribes of the Khereksur and Deer Stone culture interacted with the population of other regions, including remote ones (China, the Middle East, and North Asia) [16]. A recent study, based on an analysis of complete mitochondrial genome sequences, showed that the Ushkin-Uver archaeological site horses (9th to mid-8th century BC) of the same culture are closely related to earlier Chalcolithic Botai culture horses (mid-4th millennium BC) of the North Asian steppe and horses of Moldova (15th to 11th century BC, Mciurin site) and Germany (late 3rd to mid-2nd millennium BC, Schloßvippach site) of the Bronze Age [4]. On the basis of the above information, it cannot be presumed that the mitotypes of the Ushkin-Uver horses are ancestral for related mitotypes, especially because a complete nuclear genome analysis has revealed the origin of these horses from the horses of the South Ural (Russia) Sintashta culture of the Bronze Age (2nd millennium BC) [4]. The Ganga Tsagaan ereg horses belong to an earlier time period (late 12th to mid-10th century BC) than do Ushkin-Uver horses (9th to mid-8th century BC). We did not use the mitogenome consensus sequences of Ushkin-Uver ancient horses in the phylogeographic analysis because they had not been uploaded to the nucleotide databases. If we take into account the data obtained from the Ushkin-Uver horses, it does not seem correct to draw final conclusions about the ancestry of ancient Ganga Tsagaan ereg horse mitotypes toward the similar mitotypes of modern horses because it is likely that they were introduced to Mongolia in an earlier epoch.

Given that we found some continuity between the Mongolian horse populations of the Khereksur and Deer Stone culture and the Xiongnu culture, the occurrence of related mitotypes among the horse populations of the native breeds of China, the Middle East, and Italy can be explained by the relationship between the Xiongnu Empire and the Han Empire of China as well as the Roman empire. Some historical and archaeological data also suggest the continuity between the Khereksur and Deer Stone culture and the Xiongnu culture. However, which archaeological culture is ancestral in relation to the Xiongnu has not been identified yet [18]. What unites the cultures of the Khereksur and Deer Stone and the Xiongnu is the ritual burial of heads and hooves of horses, sheep, goats, cattle, camels, and dogs [54].

Much historical and archaeological evidence indicates that horse domestication took place in Central Asia, from where domesticated horses were distributed to other regions. Their introgression with wild horse populations in Mongolia and subsequent migration southward to China at the end of the 2nd millennium BC is assumed [55]. Around the 12th century BC, during the existence of the Chinese Shang state, when the Khereksur and Deer Stone culture was being formed on the territory of Mongolia, domesticated horses and horse-drawn carriages became widespread throughout Central China, as evidenced by written sources and the presence of horse bones in the corresponding burials [56]. In the next epoch of the Western Zhou (late 11th to early 8th century BC), there are reports of military clashes with the armed cavalry of the northern nomads. At all stages of the relationship between the Xiongnu Empire and the Han Empire (war, peaceful coexistence, including cross-border trade, gift giving, and payment of tributes), horses were exchanged, and a larger number of horses were exported from the Xiongnu to the Han Empire, not vice versa [18]. However, in the 3rd to 2nd century BC, the Chinese Empire did not have enough horses for its army. To increase the mobility of the troops, the Chinese people began to breed horses brought by Chinese merchants and travelers from the Fergana Valley [57,58,59]. From the above information, we can conclude that there are several distribution pathways of the horse mitotypes being considered: disengaged migration of ancient horses from Central Asia to China and Mongolia, migration to Mongolia via China, or to China via Mongolia. It is difficult to find out which of the considered hypotheses is the most correct based only on the data available.

Archaeological evidence also confirms the existence of long-distance contacts between the Xiongnu Empire and the Roman Empire. In the elite burial mound of the Noin-Ula site in Northern Mongolia, an antique silver plate has been unearthed portraying goddess Artemis and a satyr; this plate must have been one of horse harness parts [60]. Furthermore, a cup made of Roman glass has been discovered in the Xiongnu cemetery Gol Mod 2 of Northern Mongolia [61]. According to results of a polymorphism analysis of mitochondrial D-loop hypervariable region 1 (HV1), it has been determined that a buried person from a large settlement of Roman province Vagnari in Southern Italy belongs to a haplogroup originating from East Asia [62]. Findings in several studies reveal close genetic relationships between the mitotypes of Xiongnu culture ancient horses and indigenous horses of the Mediterranean region [4,63]. A molecular genetic study analyzing partial sequences of mitogenomes has shown a close phylogenetic relationship between ancient horses of Xiongnu royal tomb complex Tsaram (the Republic of Buryatia, Russia) and a modern horse of native Italian breed Maremmano [63], which was developed by the Etruscans in the 8th century BC [64]. An analysis of whole-mitogenome sequences has revealed that some Xiongnu horses were related to the horses from the territory of France dating to the Gallo-Roman period of classical antiquity (site of “Boulevard de la Courtille C277”) [4]. The consensus sequences of ancient horse mitogenomes obtained in the above studies were not used in our phylogeographic analysis because some sequences represent only a partial mitochondrial genome, while others had not been uploaded to the nucleotide databases.

The existence of related mitotypes in the ancient Mongolian horse populations of the Khereksur and Deer Stone culture and the Xiongnu culture indicates certain continuity of the horse populations of these cultures. The presence of similar mitotypes in the modern horse populations of breeds indigenous to Eastern and Central Asia, the Middle East, and the Mediterranean region may reflect migration routes of the ancient horse populations associated with various contacts between the Xiongnu Empire, Han Empire, and Roman Empire. Archaeological, historical, and molecular genetic data indicate that the direction of domesticated-horse mitotype migration is likely to have been from Central Asia to other regions. Nonetheless, to identify the directions more precisely, it is necessary to conduct genetic studies involving a larger number of ancient horse samples of various archaeological cultures and to include nuclear markers.

### 4.3. Population Dynamics of the Mongolian Horse Breed

As shown in the constructed phylogenetic tree (Figure 1), the majority of modern and historical (20th century) horses of the Mongolian breed are affiliated with the same haplogroups as the ancient Mongolian horses studied here. Only one modern Mongolian horse was found to be exceptional and belongs to haplogroup A, and two other modern horse haplotypes are located in distant tree branches and diverged from other haplogroups 70–145 thousand years ago (median age estimates). The ancient Mongolian horse populations being investigated were found not to possess these basal haplotypes, which seems unusual. Although several studies involving an analysis of the mitogenome hypervariable-region sequences have also revealed higher mitochondrial diversity of modern Mongolian horse populations, the following mitotypes have been identified in ancient populations: A, D2, E, and X2b [12,13], while in modern ones: B2, D3, I, K, K2a, K2b, K3, X2, X3, and X3c1, according to the classification of Cieslak et al. [12]. This result may be due to a larger sample set size of the modern horses used in the research studies. To determine the actual genetic diversity of ancient Mongolian horse populations, further research on ancient DNA including whole-genome analysis is needed.

## 5. Conclusions

Our results revealed some continuity in a mitochondrial haplotype pattern between the Khereksur and Deer Stone Mongolian horse population and the Xiongnu Mongolian horse population. In the context of possible migration routes, we determined the mitotype kinship of the investigated ancient Mongolian horses and of the modern indigenous ones from Eastern and Central Asia, the Middle East, and the Mediterranean region. For more correct assessments of genetic diversity, continuity, and migration directions of horse populations from different archaeological cultures, further ancient-DNA studies are needed.

## Figures and Tables

**Table 1:** The main characteristics of the mitochondrial genomes of the ancient horses of Mongolia.

Sample Name	Number of Collapsed Reads	Number of Unique Mapped Collapsed Reads	Mitogenome Width of Coverage, %	Mitogenome Average Depth of Coverage	Ancient Library Average Fragment Size	Terminal Library Fragment Deamination, %
Er1	188,493	917	97.3	6.7	90	14.49
Gan1	714,656	3143	99.1	18.6	60	24.31
Gan3	743,175	6919	99.8	41.8	63	25.88
Gan11	549,992	13,893	99.7	85.9	68	28.51
Gan14	511,882	11,643	99.8	66.7	65	33.77
Gan18	582,595	3322	99.5	18.5	59	25.26

**Table 2:** BEAST age estimates of the relevant nodes with the ancient Mongolian horses studied in the phylogenetic tree constructed here.

Node Name	Median Age Estimate	95% HPD (Height Posterior Density)
Er1_Arabian_Akhal-Teke	3885.2	[2330.9; 6645.42]
Gan11_Giara_Naqu	5056.25	[3105.38; 7914.36]
Gan14_Iranian_Tengchong_Yunnan_Tengchong	5687.42	[3150.41; 9072.17]
Gan18_Jinjiang	4432.33	[2853.16; 7625.08]
Gan1_Gan3_Caspian	9416.83	[5911.57; 14,467.8]

## Data Availability

The data presented in this study are openly available in [Sequence Read Archive database (SRA), GenBank database], reference numbers [PRJNA694234, MW534078, MW534079, MW534080, MW534081, MW534082, MW534083].

## Supplementary Materials

The following are available online at https://www.mdpi.com/2073-4425/12/3/412/s1, Figure S1: Ancient DNA damage patterns, Figure S2: Read length distribution plot for the ancient sample libraries, Figure S3: Visualization of the coverage depth of the obtained mitogenomes of ancient Mongolian horses, Figure S4: Initial phylogenetic tree (with *Equus asinus*) built here showing the 95% height posterior density of age estimates, Figure S5: Initial phylogenetic tree (without *Equus asinus*) built here showing the 95% height posterior density of age estimates, Table S1: Information about bone specimens of the studied ancient horses, Table S2: Information about the mitogenome sequences from GenBank.
