# Supplementary material for: Traces of Late Bronze and Early Iron Age Mongolian Horse Mitochondrial Lineages in Modern Populations
Source: Genes (Basel). 2021 Mar 12;12(3):412. doi: 10.3390/genes12030412 (PMC8000342; doi:10.3390/genes12030412)

Nucleotide mis-incorporation plot for sample Er1

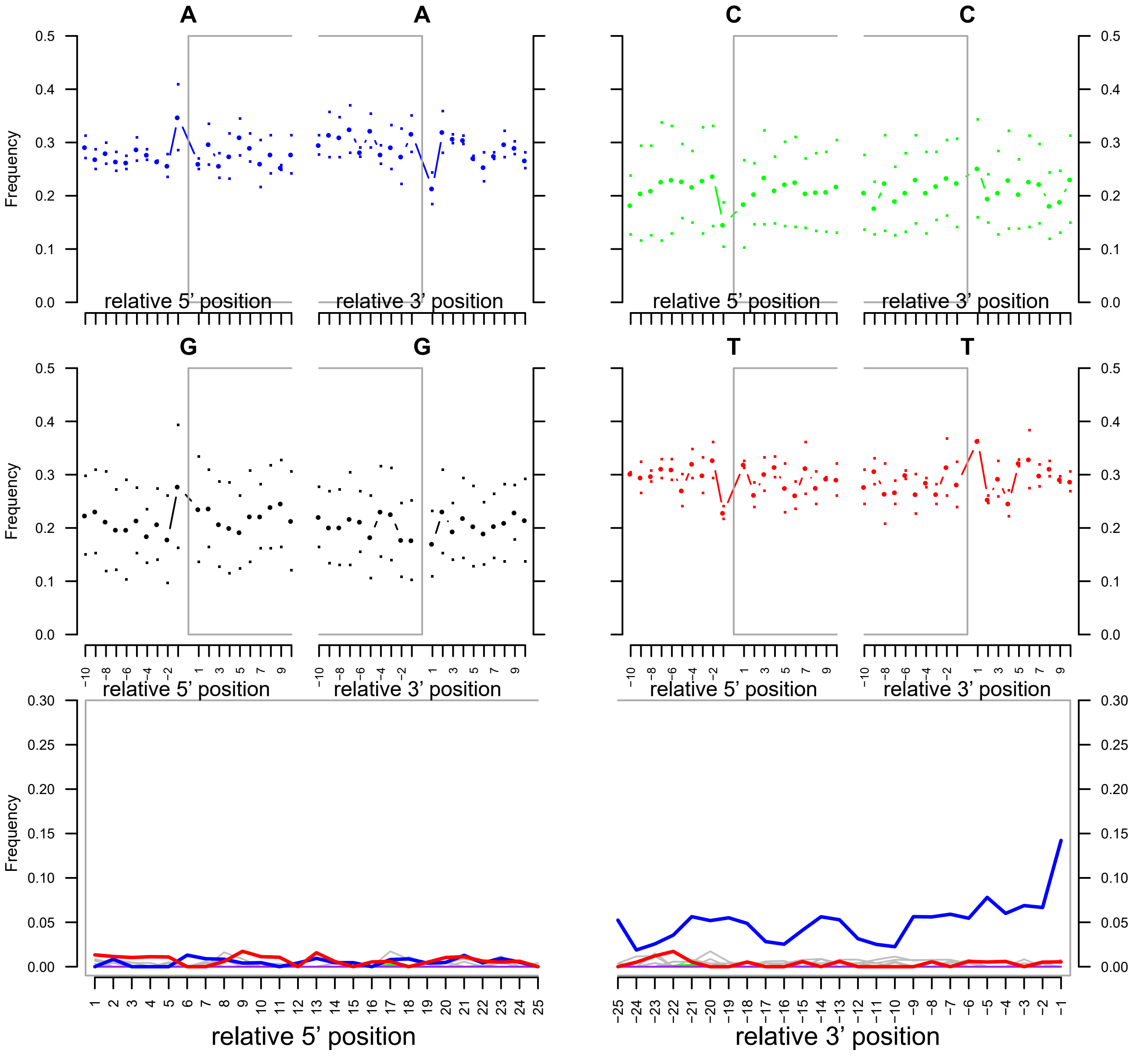

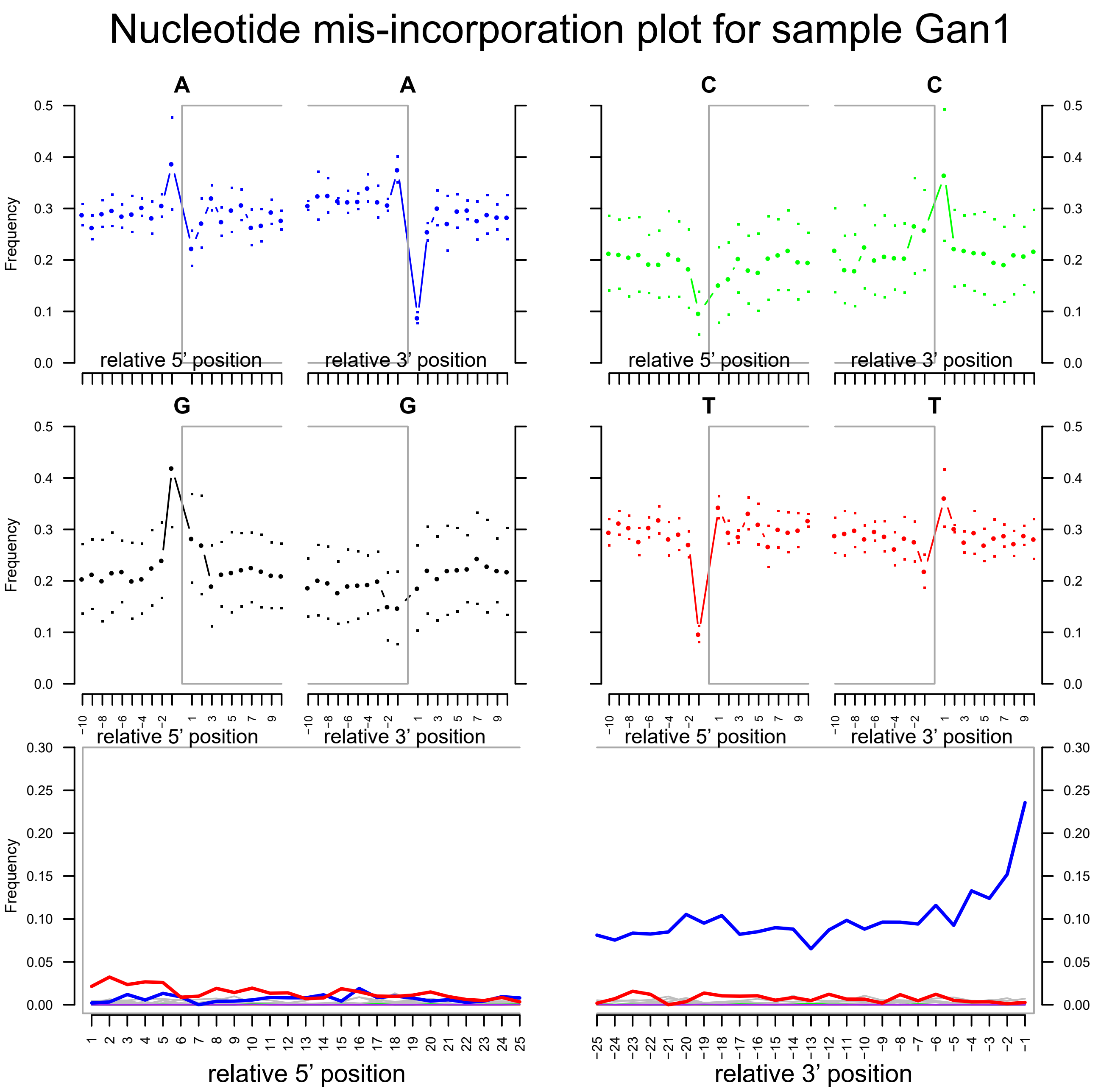

Nucleotide mis-incorporation plot for sample Gan1
A
A
C
C
G
G
T
T
Frequency
relative 5' position
relative 3' position

Nucleotide mis-incorporation plot for sample Gan3

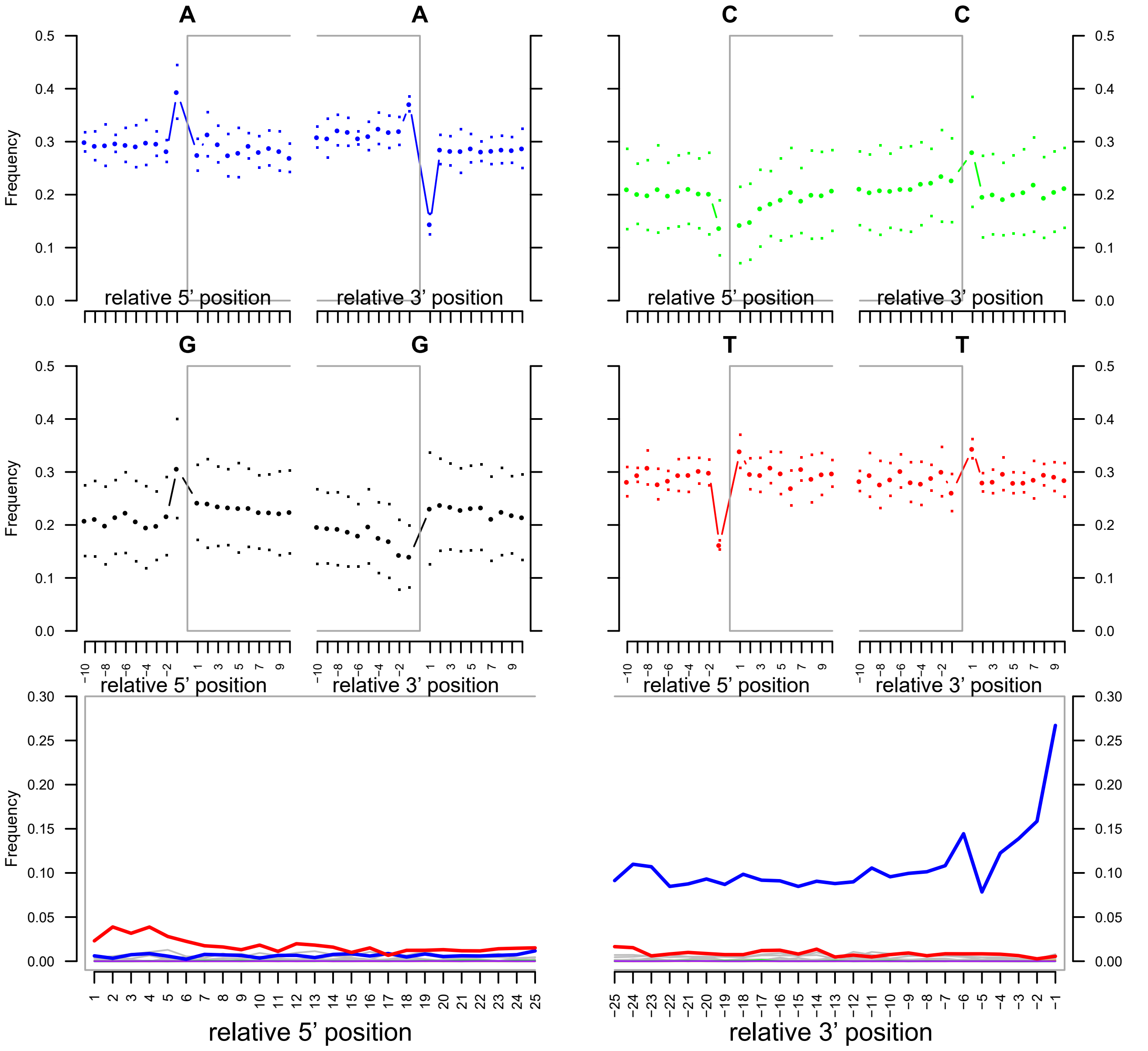

Nucleotide mis-incorporation plot for sample Gan11

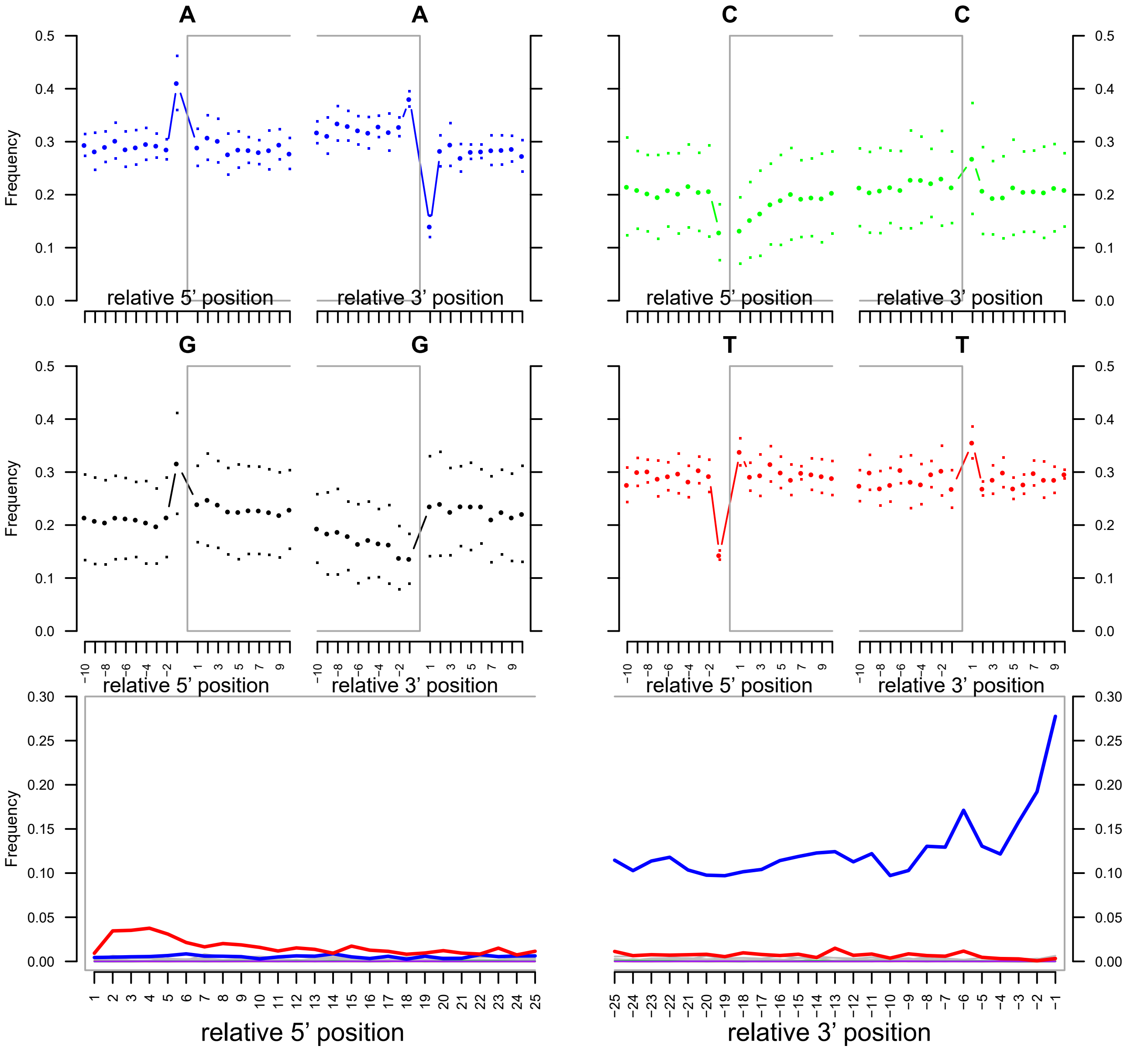

Nucleotide mis-incorporation plot for sample Gan14

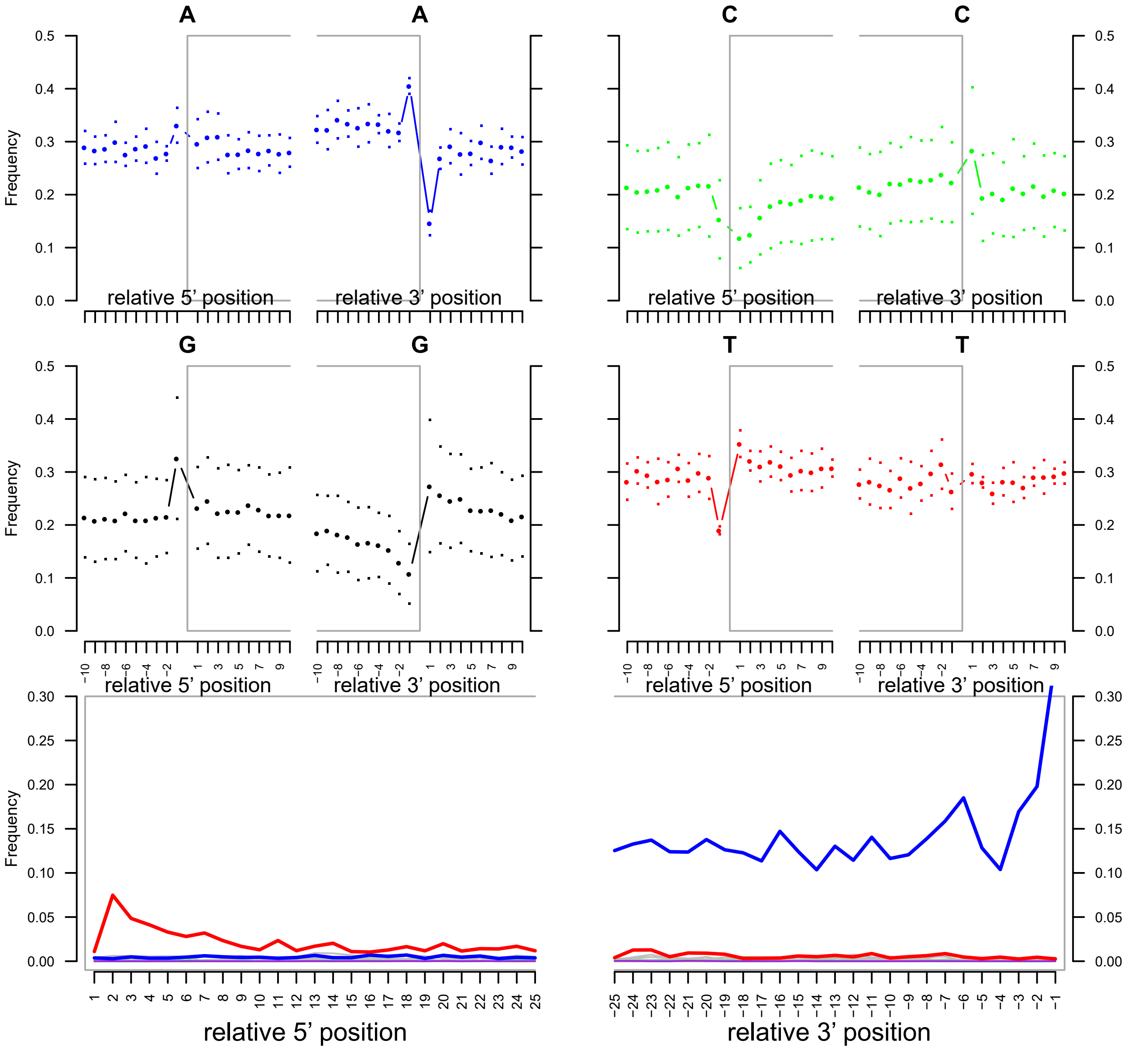

Nucleotide mis-incorporation plot for sample Gan18

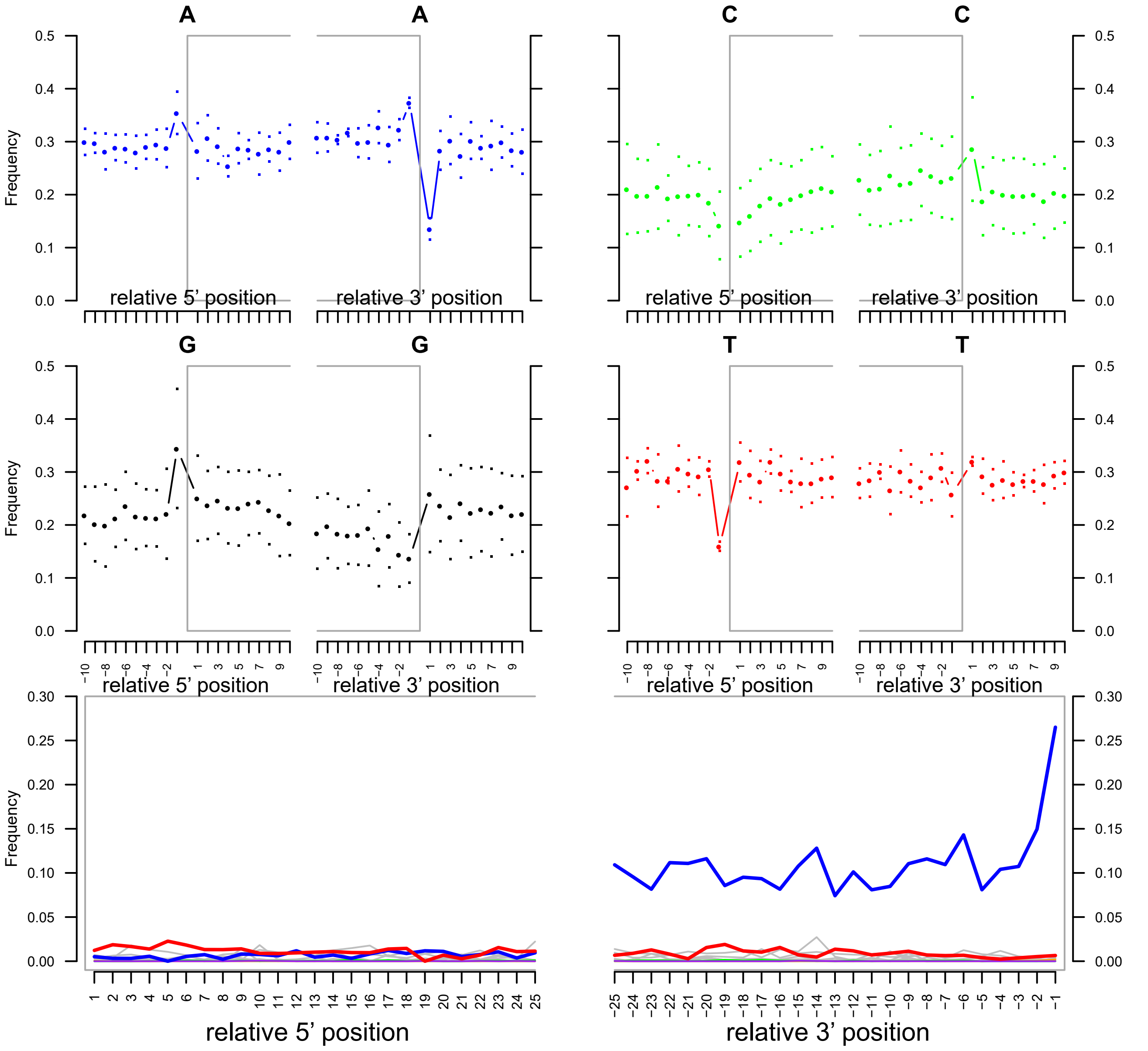

Supplement: Supplementary file 1 [file genes-12-00412-s001.zip › Figure S1. Ancient DNA damage patterns..pdf]
