# Supplementary material for: Traces of Late Bronze and Early Iron Age Mongolian Horse Mitochondrial Lineages in Modern Populations
Source: Genes (Basel). 2021 Mar 12;12(3):412. doi: 10.3390/genes12030412 (PMC8000342; doi:10.3390/genes12030412)

Read length distribution plot for sample Er1

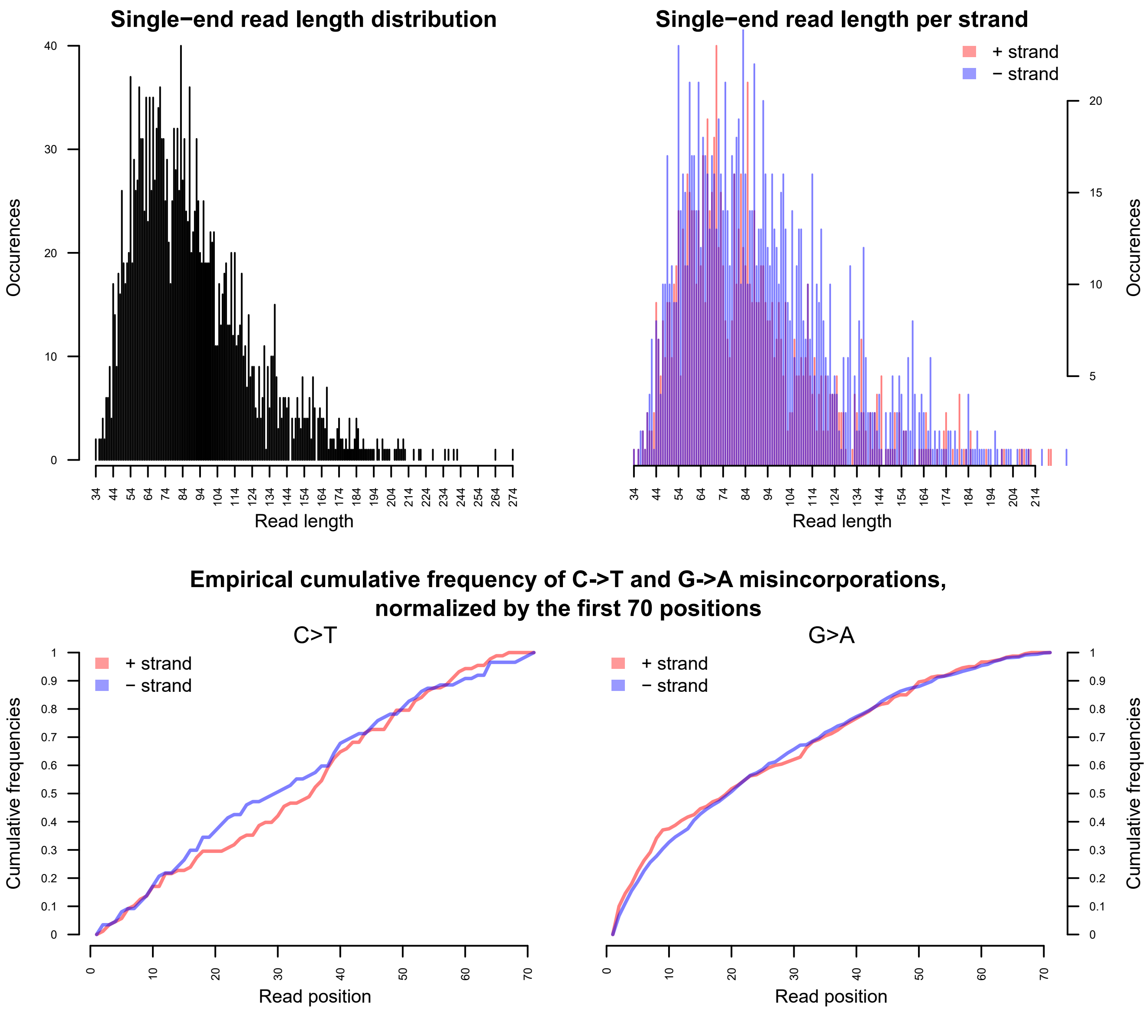

Read length distribution plot for sample Gan1

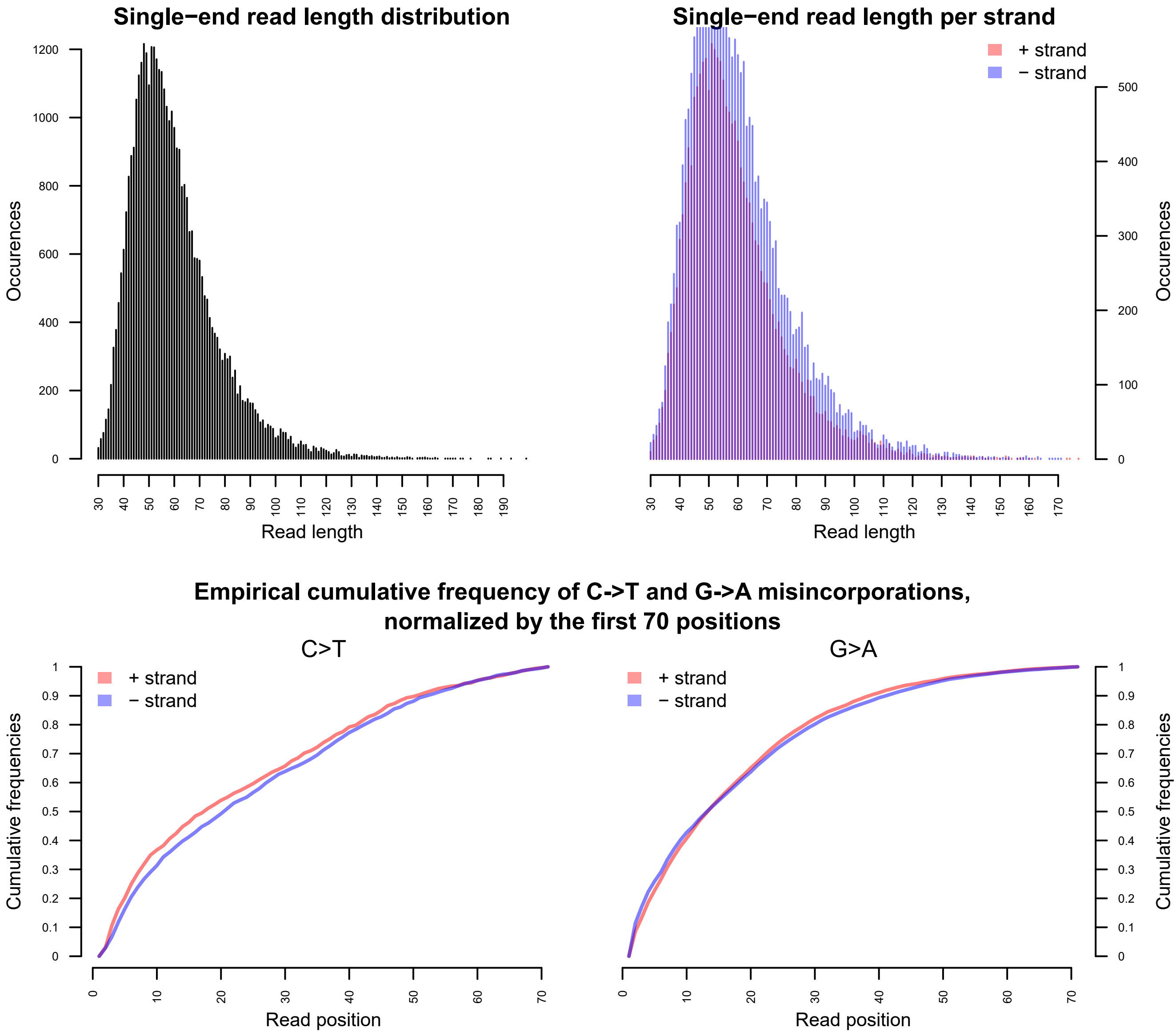

Read length distribution plot for sample Gan3

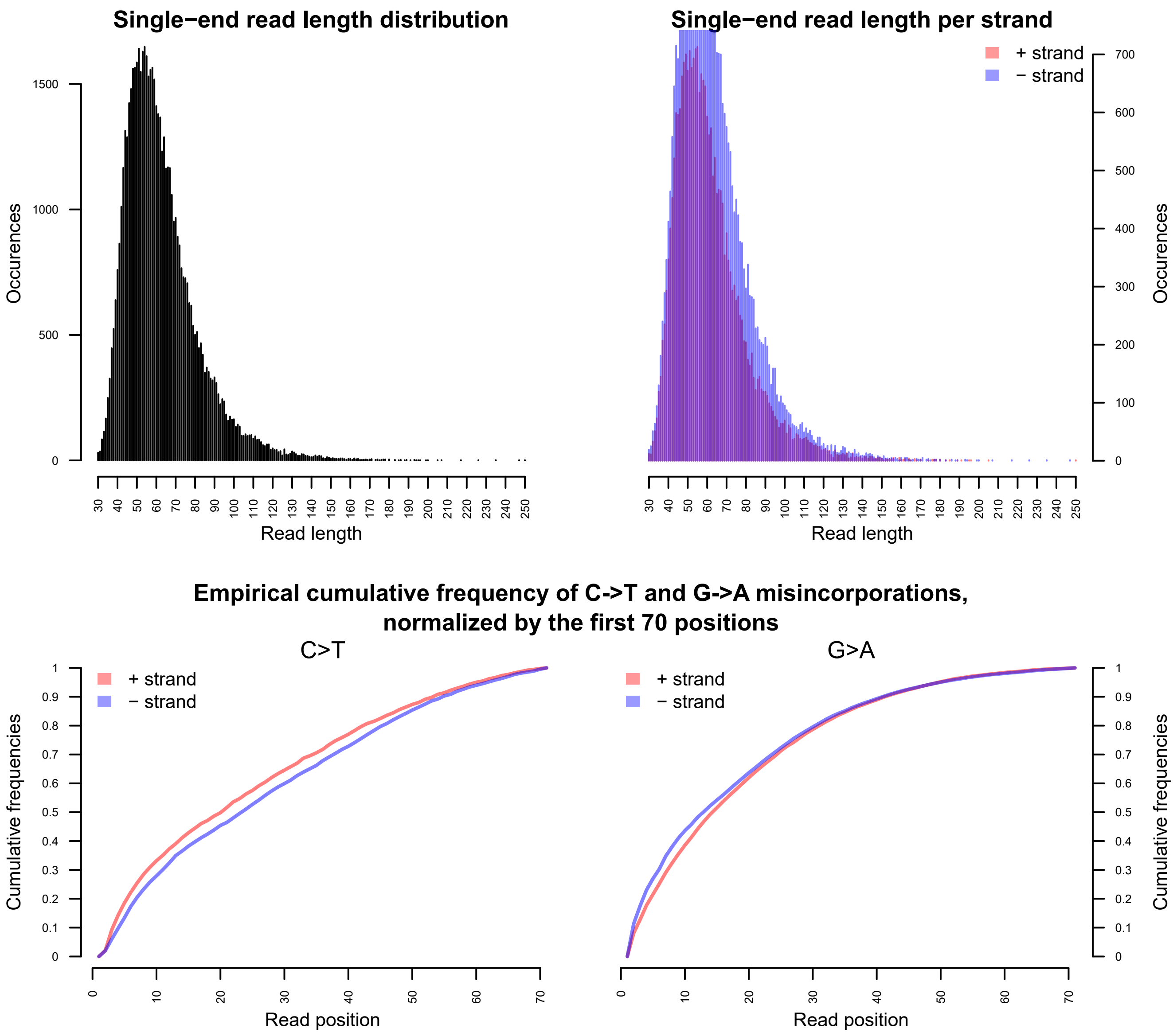

Read length distribution plot for sample Gan11

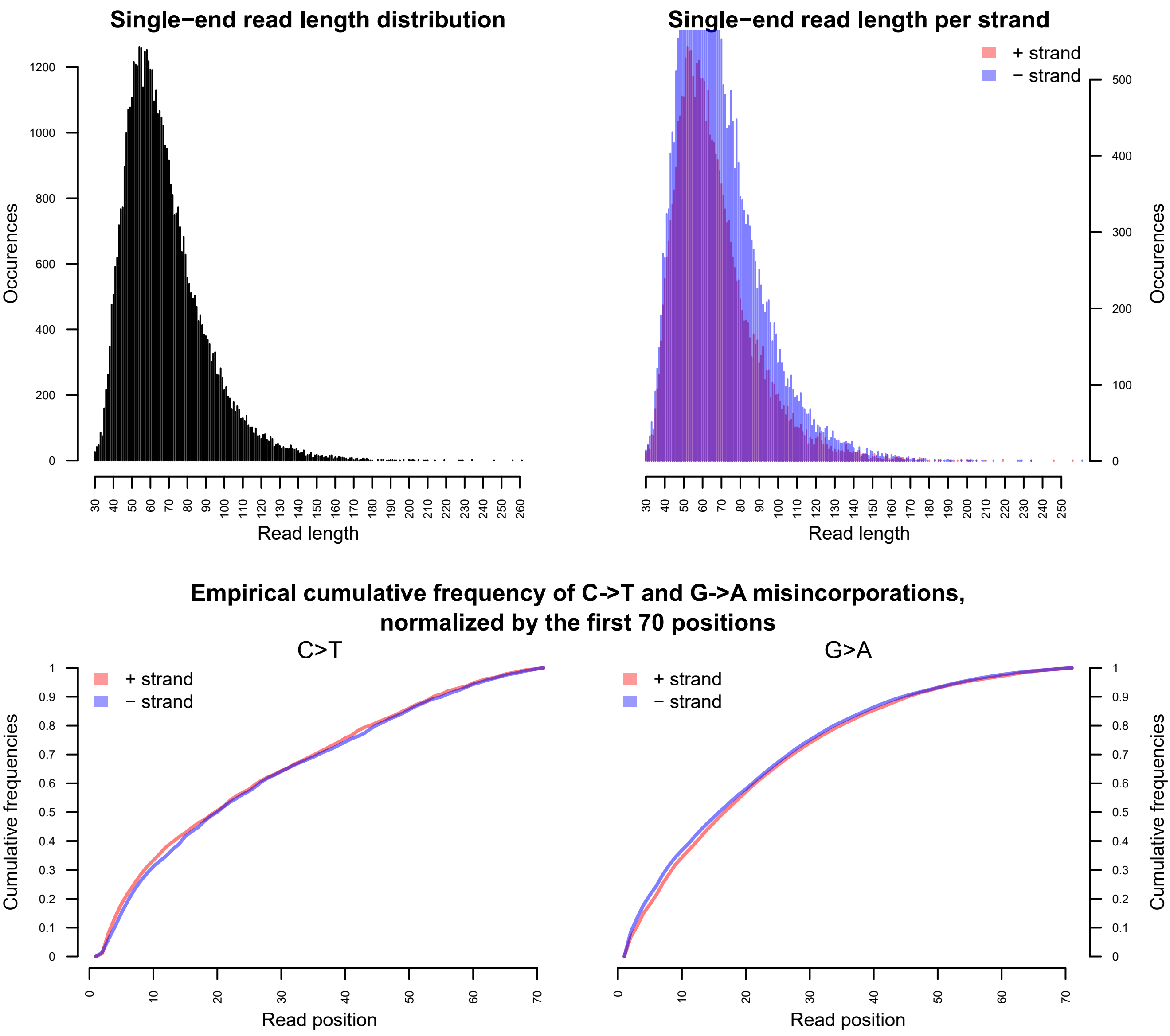

Read length distribution plot for sample Gan14

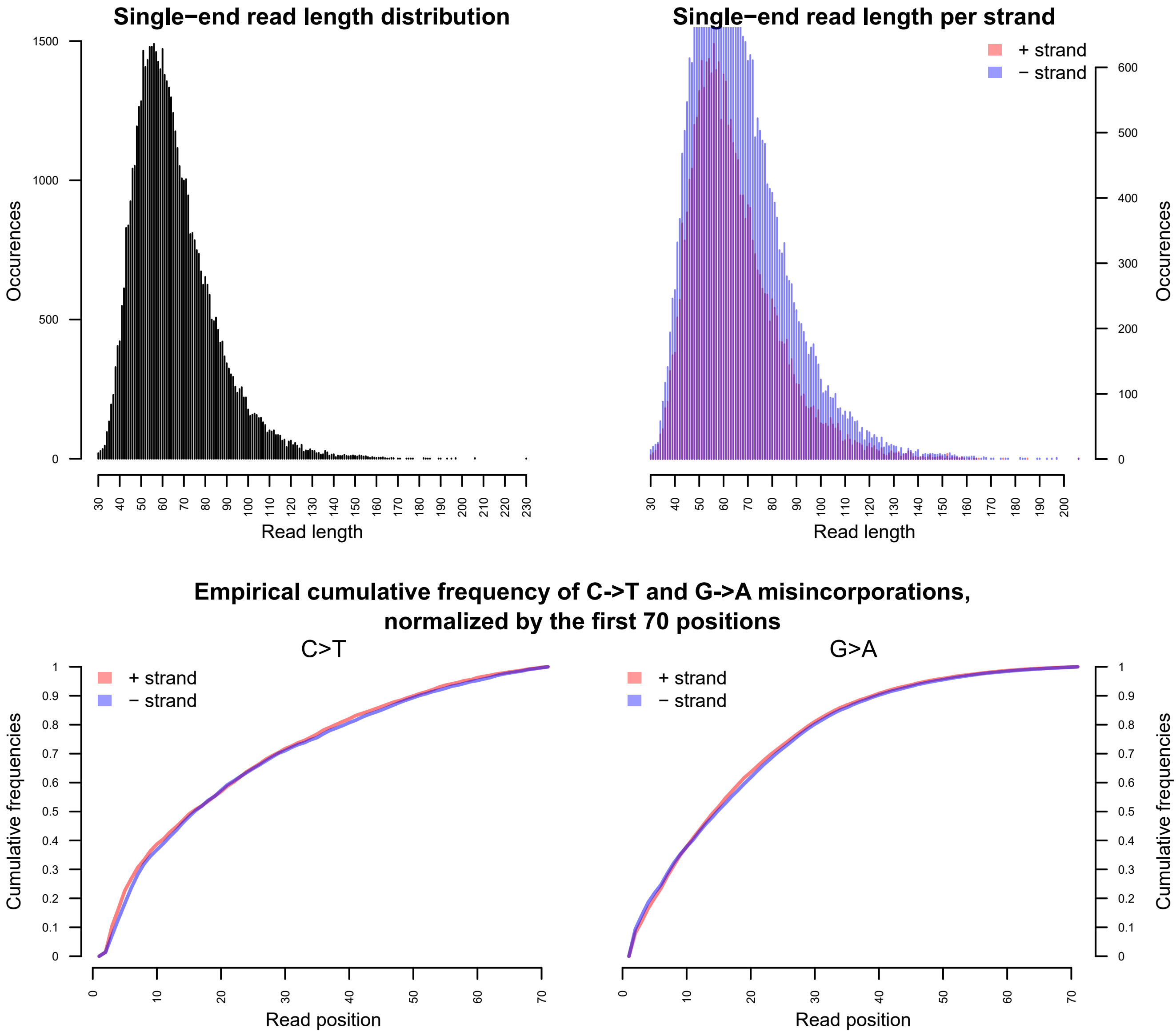

Read length distribution plot for sample Gan18

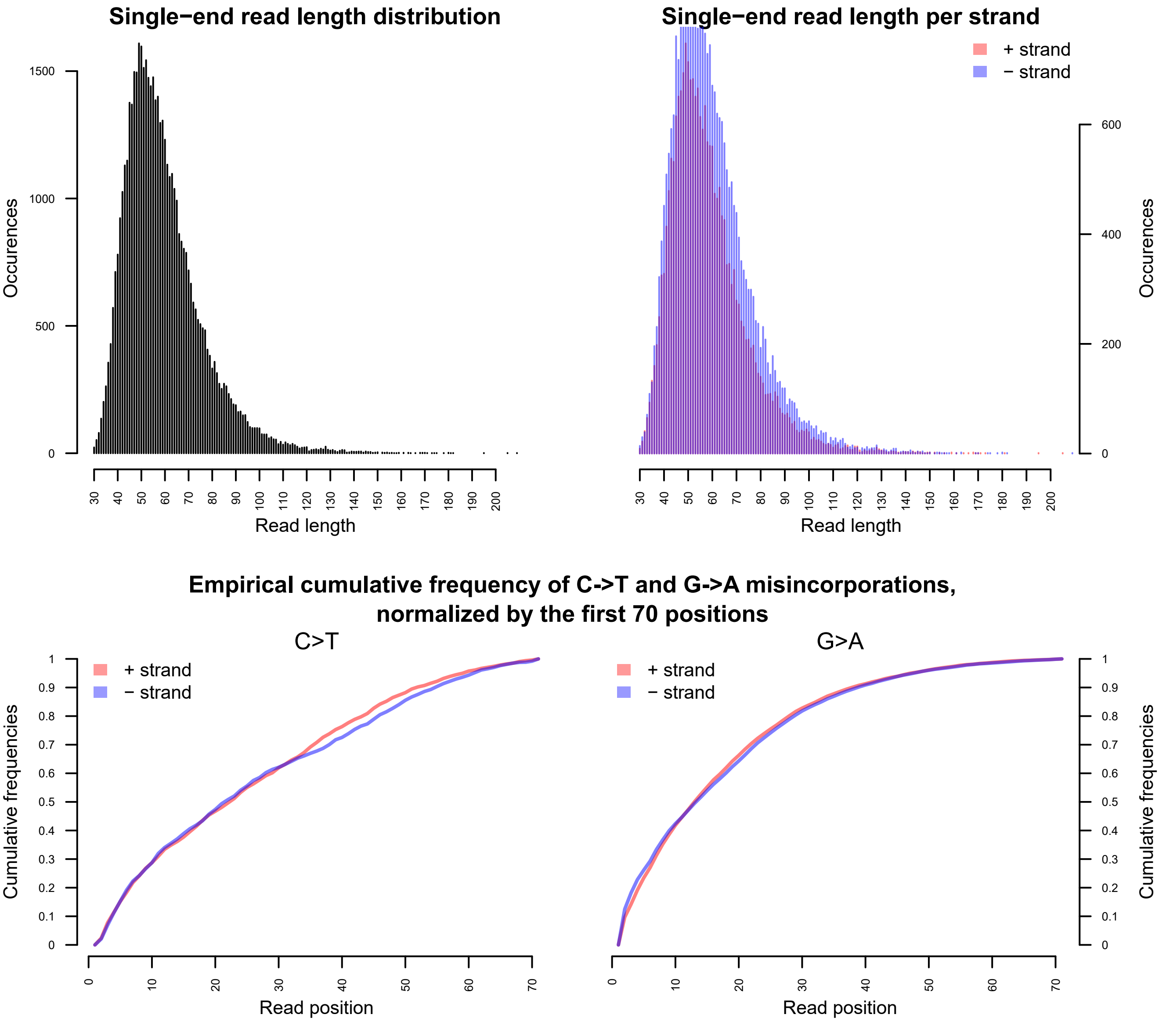

Supplement: Supplementary file 1 [file genes-12-00412-s001.zip › Figure S2. Read length distribution plot for the ancient sample libraries..pdf]
