# Supplementary material for: Traces of Late Bronze and Early Iron Age Mongolian Horse Mitochondrial Lineages in Modern Populations
Source: Genes (Basel). 2021 Mar 12;12(3):412. doi: 10.3390/genes12030412 (PMC8000342; doi:10.3390/genes12030412)
Supplement: Supplementary file 1 [file genes-12-00412-s001.zip › Figure S3. Visualization of the coverage depth of the obtained mitogenomes of ancient Mongolian horses..pdf]

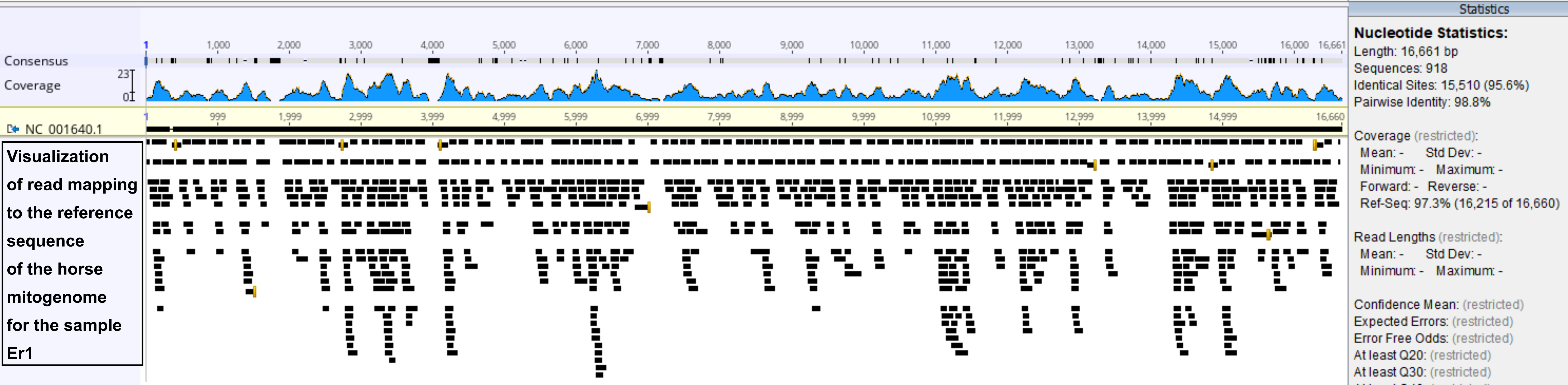

Visualization of read mapping to the reference sequence of the horse mitogenome for the sample Er1

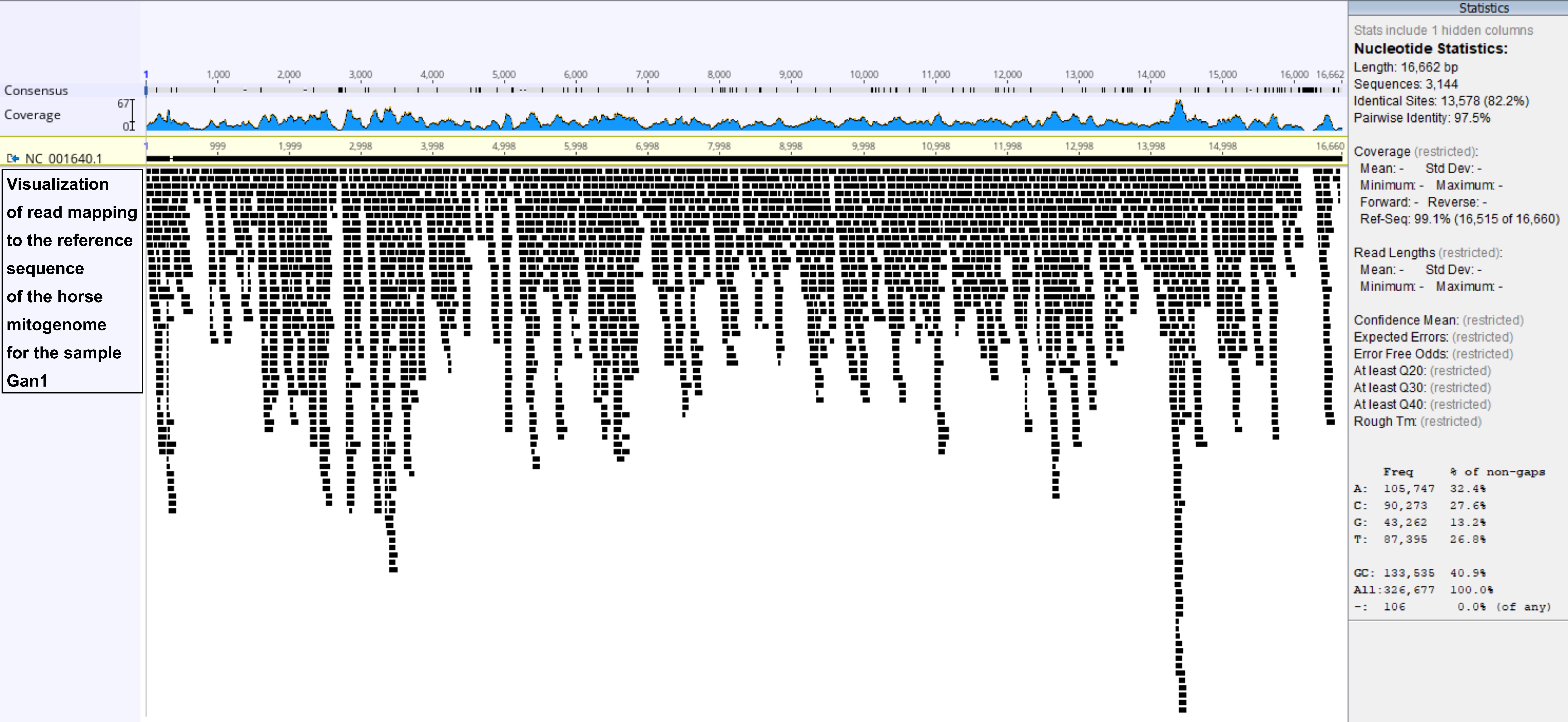
Consensus
Coverage
67
0
NC_001640.1
Visualization of read mapping to the reference sequence of the horse mitogenome for the sample Gan1
Statistics
Stats include 1 hidden columns
Nucleotide Statistics:
Length: 16,662 bp
Sequences: 3,144
Identical Sites: 13,578 (82.2%)
Pairwise Identity: 97.5%
Coverage (restricted):
Mean: - Std Dev: -
Minimum: - Maximum: -
Forward: - Reverse: -
Ref-Seq: 99.1% (16,515 of 16,660)
Read Lengths (restricted):
Mean: - Std Dev: -
Minimum: - Maximum: -
Confidence Mean: (restricted)
Expected Errors: (restricted)
Error Free Odds: (restricted)
At least Q20: (restricted)
At least Q30: (restricted)
At least Q40: (restricted)
Rough Tm: (restricted)
Freq % of non-gaps
A: 105,747 32.4%
C: 90,273 27.6%
G: 43,262 13.2%
T: 87,395 26.8%
GC: 133,535 40.9%
All: 326,677 100.0%
-: 106 0.0% (of any)

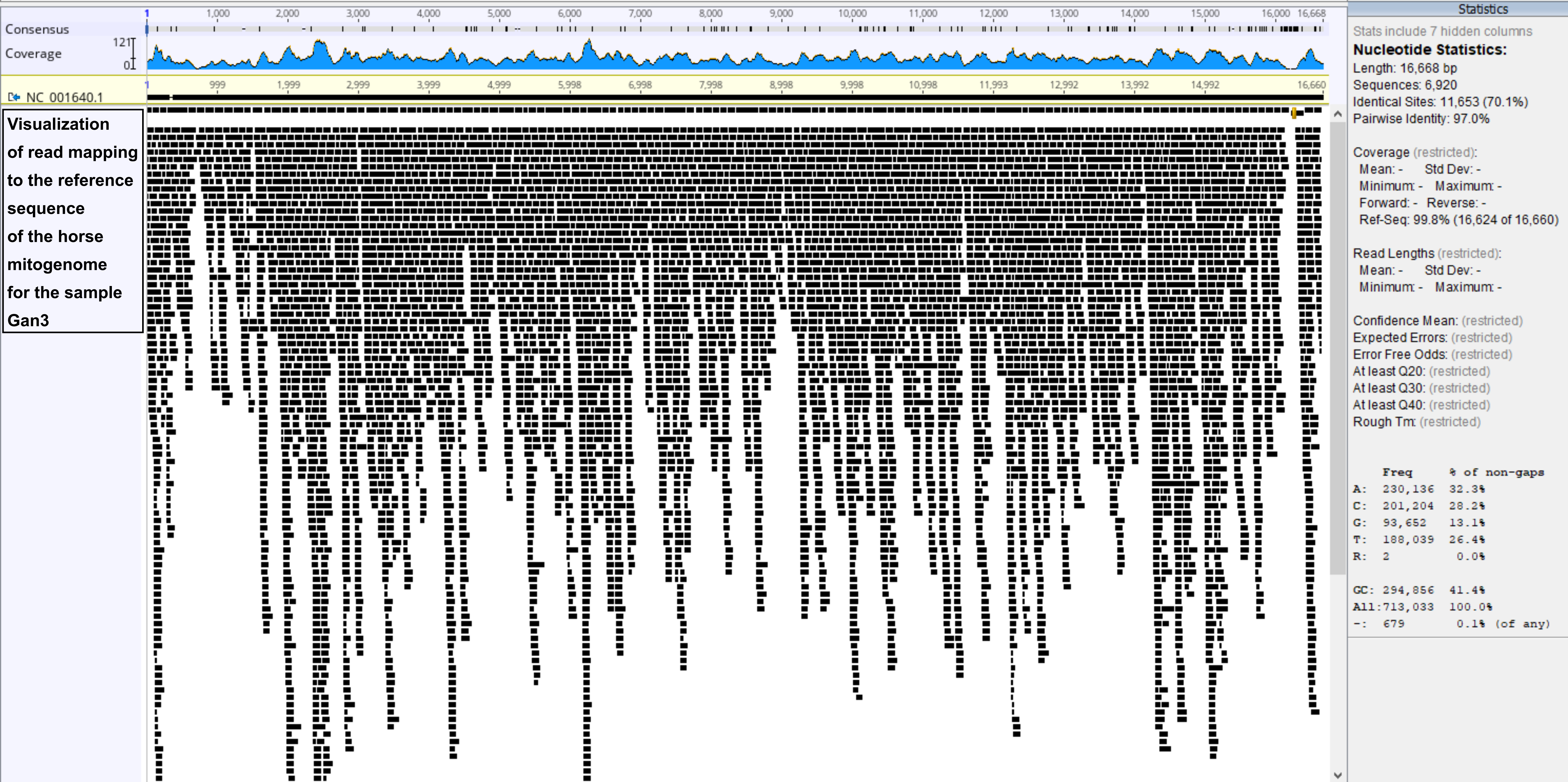

Consensus
Coverage
121
0
NC_001640.1
Visualization of read mapping to the reference sequence of the horse mitogenome for the sample Gan3
Statistics
Stats include 7 hidden columns
Nucleotide Statistics:
Length: 16,668 bp
Sequences: 6,920
Identical Sites: 11,653 (70.1%)
Pairwise Identity: 97.0%
Coverage (restricted):
Mean: - Std Dev: -
Minimum: - Maximum: -
Forward: - Reverse: -
Ref-Seq: 99.8% (16,624 of 16,660)
Read Lengths (restricted):
Mean: - Std Dev: -
Minimum: - Maximum: -
Confidence Mean: (restricted)
Expected Errors: (restricted)
Error Free Odds: (restricted)
At least Q20: (restricted)
At least Q30: (restricted)
At least Q40: (restricted)
Rough Tm: (restricted)
Freq % of non-gaps
A: 230,136 32.3%
C: 201,204 28.2%
G: 93,652 13.1%
T: 188,039 26.4%
R: 2 0.0%
GC: 294,856 41.4%
All: 713,033 100.0%
-: 679 0.1% (of any)

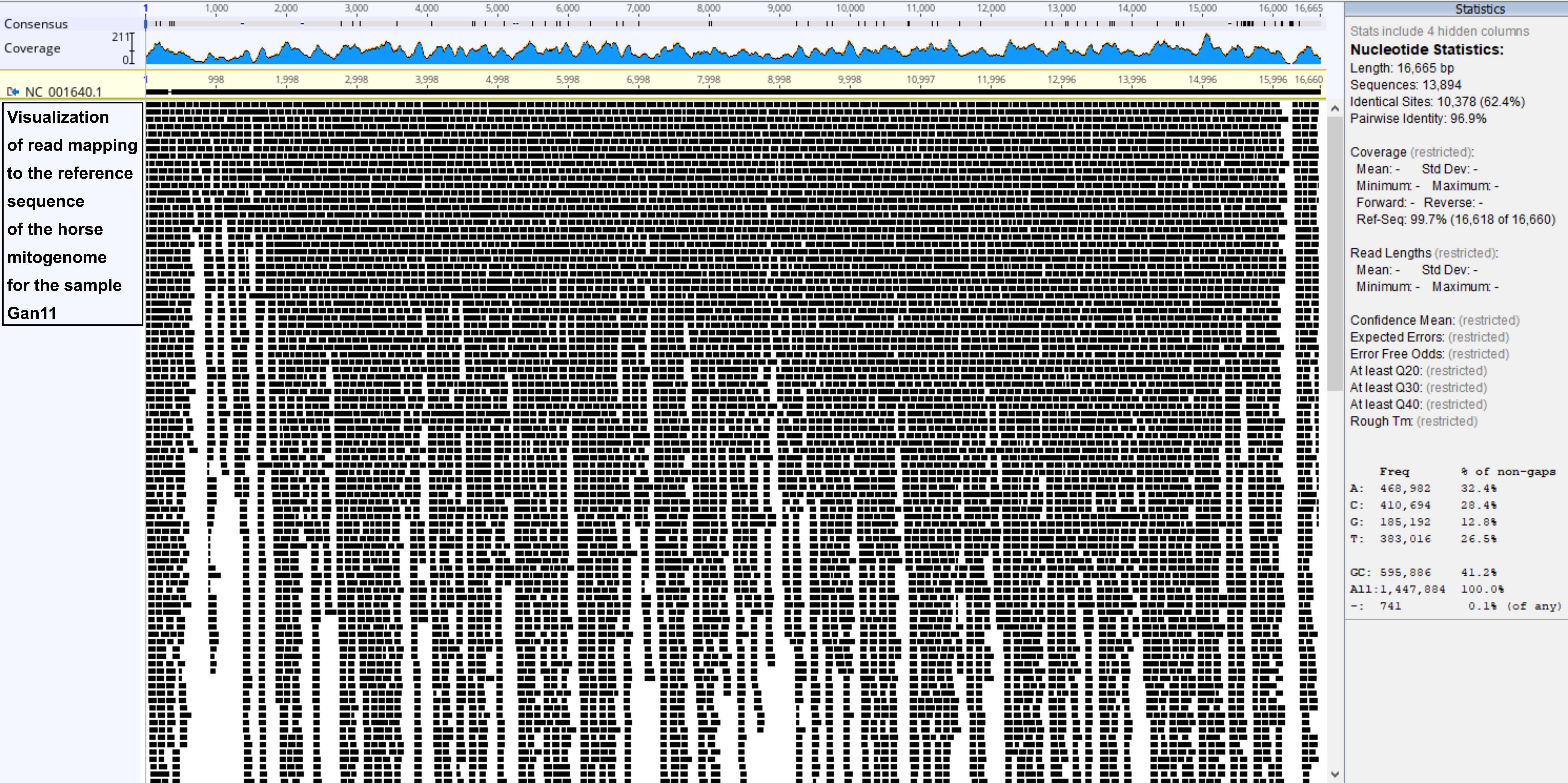
Consensus
Coverage
211
0
NC_001640.1
1
1,000
2,000
3,000
4,000
5,000
6,000
7,000
8,000
9,000
10,000
11,000
12,000
13,000
14,000
15,000
16,000
16,665
998
1,998
2,998
3,998
4,998
5,998
6,998
7,998
8,998
9,998
10,997
11,996
12,996
13,996
14,996
15,996
16,660
Visualization of read mapping to the reference sequence of the horse mitogenome for the sample Gan11
Statistics
Stats include 4 hidden columns
Nucleotide Statistics:
Length: 16,665 bp
Sequences: 13,894
Identical Sites: 10,378 (62.4%)
Pairwise Identity: 96.9%
Coverage (restricted):
Mean: - Std Dev: -
Minimum: - Maximum: -
Forward: - Reverse: -
Ref-Seq: 99.7% (16,618 of 16,660)
Read Lengths (restricted):
Mean: - Std Dev: -
Minimum: - Maximum: -
Confidence Mean: (restricted)
Expected Errors: (restricted)
Error Free Odds: (restricted)
At least Q20: (restricted)
At least Q30: (restricted)
At least Q40: (restricted)
Rough Tm: (restricted)
Freq % of non-gaps
A: 468,982 32.4%
C: 410,694 28.4%
G: 185,192 12.8%
T: 383,016 26.5%
GC: 595,886 41.2%
All: 1,447,884 100.0%
-: 741 0.1% (of any)

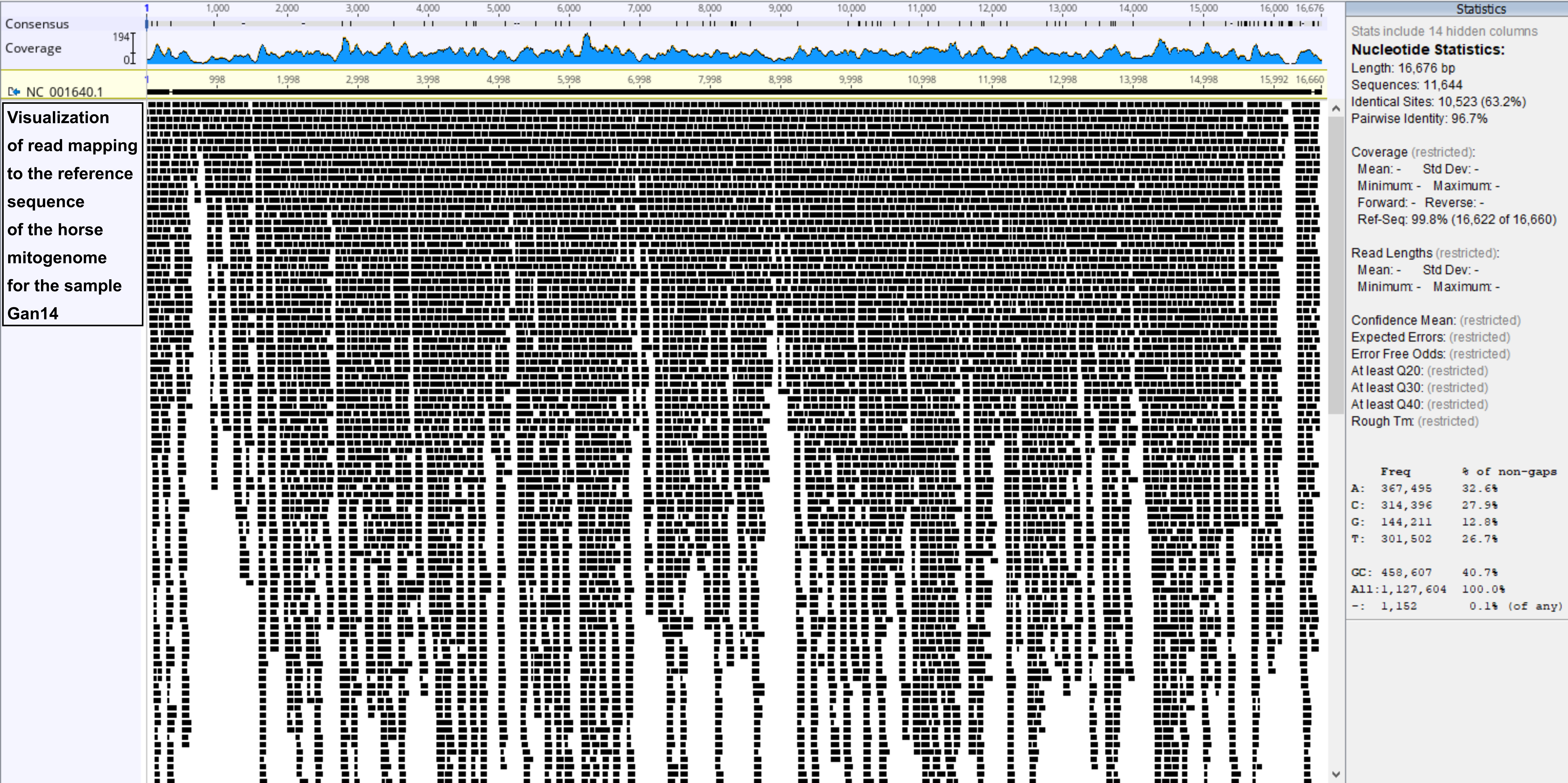

Consensus
Coverage
194
0
1
1,000
2,000
3,000
4,000
5,000
6,000
7,000
8,000
9,000
10,000
11,000
12,000
13,000
14,000
15,000
16,000
16,676
NC_001640.1
998
1,998
2,998
3,998
4,998
5,998
6,998
7,998
8,998
9,998
10,998
11,998
12,998
13,998
14,998
15,992
16,660
Visualization of read mapping to the reference sequence of the horse mitogenome for the sample Gan14
Statistics
Stats include 14 hidden columns
Nucleotide Statistics:
Length: 16,676 bp
Sequences: 11,644
Identical Sites: 10,523 (63.2%)
Pairwise Identity: 96.7%
Coverage (restricted):
Mean: - Std Dev: -
Minimum: - Maximum: -
Forward: - Reverse: -
Ref-Seq: 99.8% (16,622 of 16,660)
Read Lengths (restricted):
Mean: - Std Dev: -
Minimum: - Maximum: -
Confidence Mean: (restricted)
Expected Errors: (restricted)
Error Free Odds: (restricted)
At least Q20: (restricted)
At least Q30: (restricted)
At least Q40: (restricted)
Rough Tm: (restricted)
Freq % of non-gaps
A: 367,495 32.6%
C: 314,396 27.9%
G: 144,211 12.8%
T: 301,502 26.7%
GC: 458,607 40.7%
All: 1,127,604 100.0%
-: 1,152 0.1% (of any)

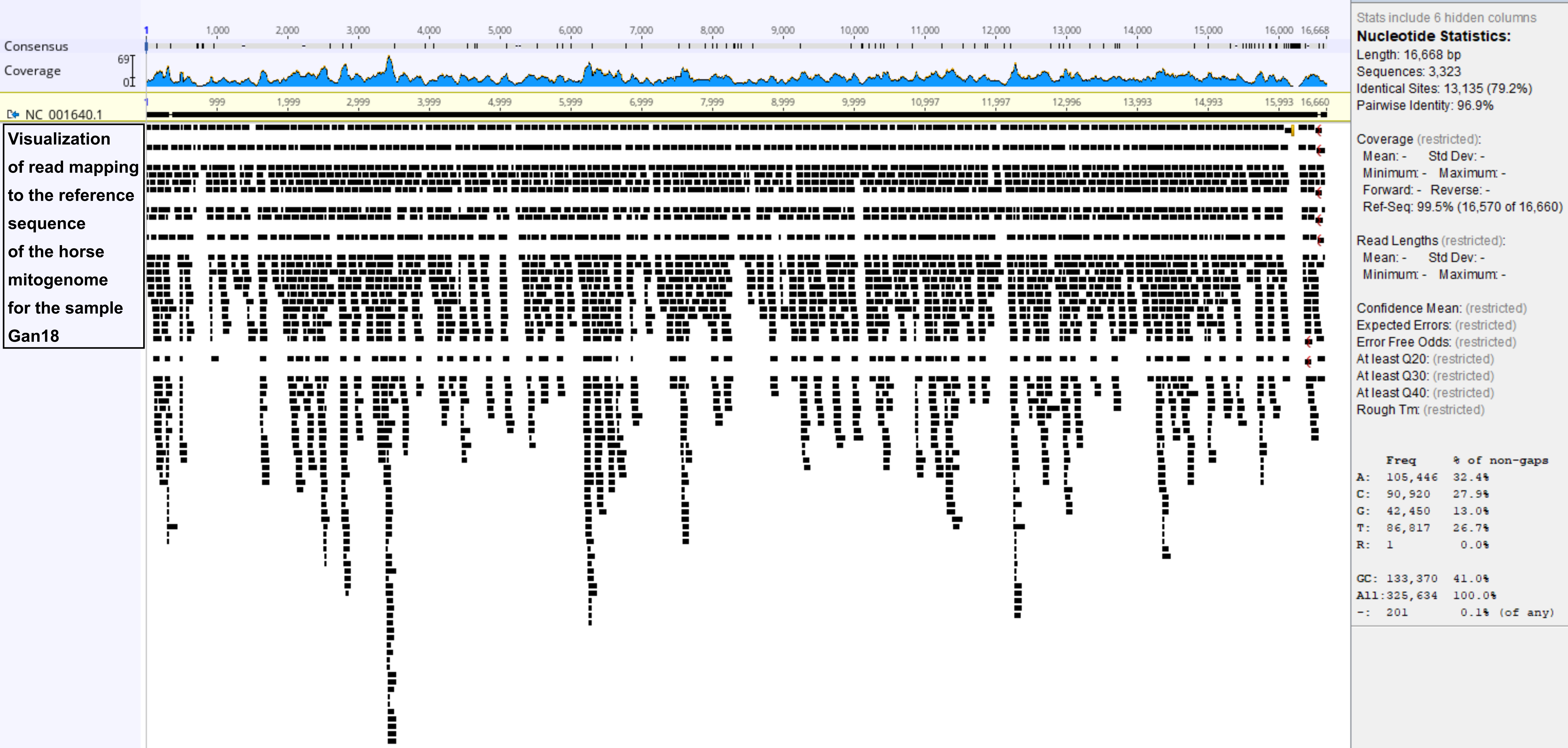
Consensus
Coverage
69
0
NC_001640.1
Visualization of read mapping to the reference sequence of the horse mitogenome for the sample Gan18
Stats include 6 hidden columns
Nucleotide Statistics:
Length: 16,668 bp
Sequences: 3,323
Identical Sites: 13,135 (79.2%)
Pairwise Identity: 96.9%
Coverage (restricted):
Mean: - Std Dev: -
Minimum: - Maximum: -
Forward: - Reverse: -
Ref-Seq: 99.5% (16,570 of 16,660)
Read Lengths (restricted):
Mean: - Std Dev: -
Minimum: - Maximum: -
Confidence Mean: (restricted)
Expected Errors: (restricted)
Error Free Odds: (restricted)
At least Q20: (restricted)
At least Q30: (restricted)
At least Q40: (restricted)
Rough Tm: (restricted)
Freq % of non-gaps
A: 105,446 32.4%
C: 90,920 27.9%
G: 42,450 13.0%
T: 86,817 26.7%
R: 1 0.0%
GC: 133,370 41.0%
All: 325,634 100.0%
-: 201 0.1% (of any)
