# Supplementary material for: Traces of Late Bronze and Early Iron Age Mongolian Horse Mitochondrial Lineages in Modern Populations
Source: Genes (Basel). 2021 Mar 12;12(3):412. doi: 10.3390/genes12030412 (PMC8000342; doi:10.3390/genes12030412)
Supplement: Supplementary file 1 [file genes-12-00412-s001.zip › Table S1. Information about bone specimens of the studied ancient horses..docx]

**Table S1.** Information about osteological specimens of the studied ancient horses.

| **Sample name** | **Sample material type** | **Archaeological site name and sample location** | **Archaeological site geographic location and GPS coordinates** | **Archaeological culture name** | **Sample age on the basis of the archaeological context** |
| --- | --- | --- | --- | --- | --- |
| Er1 | tooth | Kurgan Ereen hailaas №1-018 | Mongolia, Bulgan aimag, valley of the river Egin-Gol; 49.541831, 103.259603 | Xiongnu | The Early Iron Age, Xiongnu time, 1st century BC - 1st century AD |
| Gan1 | skull fragment (pars petrosa of the temporal bone) | Kurgan group Ganga Tsagaan ereg, altar №2 near hereksur 1-078 | Mongolia, Bulgan aimag, valley of the river Egin-Gol; 49.572897, 103.251933 | Khereksur and Deer Stone | The Late Bronze Age - Early Iron Age, Arzhan-Mayemir time,  the end of the 12th – the first half of the 10th centuries BC |
| Gan3 | tooth | Kurgan group Ganga Tsagaan ereg, altar №4 near hereksur 1-078 | Mongolia, Bulgan aimag, valley of the river Egin-Gol; 49.572897, 103.251933 | Khereksur and Deer Stone | The Late Bronze Age - Early Iron Age, Arzhan-Mayemir time, the end of the 12th – the first half of the 10th centuries BC |
| Gan11 | tooth | Kurgan group Ganga Tsagaan ereg, altar №4 near hereksur 1-079 | Mongolia, Bulgan aimag, valley of the river Egin-Gol; 49.572897, 103.251933 | Khereksur and Deer Stone | The Late Bronze Age - Early Iron Age, Arzhan-Mayemir time, the end of the 12th – the first half of the 10th centuries BC |
| Gan14 | tooth | Kurgan group Ganga Tsagaan ereg, altar №10 near hereksur 1-082 | Mongolia, Bulgan aimag, valley of the river Egin-Gol; 49.572897, 103.251933 | Khereksur and Deer Stone | The Late Bronze Age - Early Iron Age, Arzhan-Mayemir time, the end of the 12th – the first half of the 10th centuries BC |
| Gan18 | tooth | Kurgan group Ganga Tsagaan ereg, altar №14 near hereksur 1-082 | Mongolia, Bulgan aimag, valley of the river Egin-Gol; 49.572897, 103.251933 | Khereksur and Deer Stone | The Late Bronze Age - Early Iron Age, Arzhan-Mayemir time, the end of the 12th – the first half of the 10th centuries BC |
