# Supplementary material for: Traces of Late Bronze and Early Iron Age Mongolian Horse Mitochondrial Lineages in Modern Populations
Source: Genes (Basel). 2021 Mar 12;12(3):412. doi: 10.3390/genes12030412 (PMC8000342; doi:10.3390/genes12030412)
Supplement: Supplementary file 1 [file genes-12-00412-s001.zip › Table S2. Information about the mitogenome sequences from Genbank..docx]

**Table S2.** Information about the mitogenome sequences from Genbank.

| **№** | **Sequence accession number (GenBank)** | **Species affiliation** | **Ancient or modern horse** | **Horse breed** | **Geographic location** | **Geographic region** | **Sample age, yBP** |
| --- | --- | --- | --- | --- | --- | --- | --- |
| 1 | KT368723 | *Equus lenensis* | ancient | - | Yuka site, Ust-Yansky District, Northern Yakutia, Sakha Republic, Russia | Northern Asia | 5389 |
| 2 | KT368724 | *Equus ferus* | ancient | Yakut | Odjuluun site, Churapchinsky District, Central Yakutia, Sakha Republic, Russia | Northern Asia | 230 |
| 3 | KT368725 | *Equus lenensis* | ancient | - | Batagai site, Verkhoyansky District, Central Yakutia, Sakha Republic, Russia | Northern Asia | 5093 |
| 4 | KT368726 | *Equus ferus caballus* | ancient | Yakut | Tumeski site, Verkhoyansky District, Central Yakutia, Sakha Republic, Russia | Northern Asia | 105 |
| 5 | KT368739 | *Equus ferus caballus* | ancient | Mongolian | Mongolia | East Asia | 107 |
| 6 | KT368740 | *Equus ferus caballus* | ancient | Mongolian | Mongolia | East Asia | 108 |
| 7 | KT757740 | *Equus lenensis* | ancient | Taymyr | Taymyrsky Dolgano-Nenetsky District, the Taymyr Peninsula, Krasnoyarsk Krai, Russia | Northern Asia | 43073 |
| 8 | KT757741 | *Equus lenensis* | ancient | Taymyr | Taymyrsky Dolgano-Nenetsky District, the Taymyr Peninsula, Krasnoyarsk Krai, Russia | Northern Asia | 16492 |
| 9 | KT757742 | *Equus ferus* | ancient | Taymyr | Taymyrsky Dolgano-Nenetsky District, the Taymyr Peninsula, Krasnoyarsk Krai, Russia | Northern Asia | 27590 |
| 10 | KT757743 | *Equus ferus* | ancient | Taymyr | Taymyrsky Dolgano-Nenetsky District, the Taymyr Peninsula, Krasnoyarsk Krai, Russia | Northern Asia | 29115 |
| 11 | KT757744 | *Equus ferus* | ancient | Taymyr | Taymyrsky Dolgano-Nenetsky District, the Taymyr Peninsula, Krasnoyarsk Krai, Russia | Northern Asia | 29022 |
| 12 | KT757746 | *Equus ferus* | ancient | Yakut | Bulunsky District, Bolshoy Lyakhovsky Island, Central Yakutia, Sakha Republic, Russia | Northern Asia | 4250 |
| 13 | KT757747 | *Equus ferus* | ancient | Yakut | Bulunsky District, New Siberian Islands, Central Yakutia, Sakha Republic, Russia | Northern Asia | 22039 |
| 14 | KT757748 | *Equus ferus* | ancient | Yakut | Bulunsky District, New Siberian Islands, Central Yakutia, Sakha Republic, Russia | Northern Asia | 26044 |
| 15 | KT757749 | *Equus ferus* | ancient | Yakut | Bulunsky District, New Siberian Islands, Central Yakutia, Sakha Republic, Russia | Northern Asia | 35392 |
| 16 | KT757750 | *Equus ferus* | ancient | Yakut | Bulunsky District, New Siberian Islands, Central Yakutia, Sakha Republic, Russia | Northern Asia | 41906 |
| 17 | KT757754 | *Equus ferus* | ancient | Ural | Gornozavodsky District, the Ural Mountains, Perm Krai, Russia | Eastern Europe-Northern Asia | 34272 |
| 18 | KT757755 | *Equus ferus* | ancient | Ural | Gornozavodsky District, the Ural Mountains, Perm Krai, Russia | Eastern Europe-Northern Asia | 31035 |
| 19 | KT757756 | *Equus ferus* | ancient | Ural | Gornozavodsky District, the Ural Mountains, Perm Krai, Russia | Eastern Europe-Northern Asia | 35100 |
| 20 | KT757757 | *Equus ferus* | ancient | Ural | Gornozavodsky District, the Ural Mountains, Perm Krai, Russia | Eastern Europe-Northern Asia | 41456 |
| 21 | KT757758 | *Equus ferus* | ancient | Ural | Gornozavodsky District, the Ural Mountains, Perm Krai, Russia | Eastern Europe-Northern Asia | 20504 |
| 22 | KT757759 | *Equus ferus* | ancient | Ural | Gornozavodsky District, the Ural Mountains, Perm Krai, Russia | Eastern Europe-Northern Asia | 19014 |
| 23 | KT368753 | *Equus ferus przewalskii* | ancient | studbook number 11 | Mongolia | East Asia | 112 |
| 24 | KT368754 | *Equus ferus przewalskii* | ancient | studbook number 12 | Mongolia | East Asia | 110 |
| 25 | KT368755 | *Equus ferus przewalskii* | ancient | - | Dzungaria, Xinjiang Uyghur Autonomous Region, China | East Asia | 142 |
| 26 | KT368756 | *Equus ferus przewalskii* | ancient | - | The Gobi Desert, Xinjiang Uyghur Autonomous Region, China | East Asia | 121 |
| 27 | KT368758 | *Equus ferus przewalskii* | ancient | studbook number 56, hybrid | Halle, Saxony-Anhalt state, Germany | Central Europe | 93 |
| 28 | HQ439443 | *Equus ferus caballus* | modern | Altai | The Altai Republic, Russia | Northern Asia | 0 |
| 29 | HQ439444 | *Equus ferus caballus* | modern | Altai | The Altai Republic, Russia | Northern Asia | 0 |
| 30 | MG001417 | *Equus ferus caballus* | modern | Baise pony | Guangxi Zhuang Autonomous Region, China | East Asia | 0 |
| 31 | MG001445 | *Equus ferus caballus* | modern | Baise pony | Guangxi Zhuang Autonomous Region, China | East Asia | 0 |
| 32 | MG001447 | *Equus ferus caballus* | modern | Baise pony | Guangxi Zhuang Autonomous Region, China | East Asia | 0 |
| 33 | KU575247 | *Equus ferus caballus* | modern | Chakouyi | Gansu Province, China | East Asia | 0 |
| 34 | MG001426 | *Equus ferus caballus* | modern | Chakouyi | Gansu Province, China | East Asia | 0 |
| 35 | MG001444 | *Equus ferus caballus* | modern | Chakouyi | Gansu Province, China | East Asia | 0 |
| 36 | MG001416 | *Equus ferus caballus* | modern | Debao pony | Guangxi Zhuang Autonomous Region, China | East Asia | 0 |
| 37 | EU939445 | *Equus ferus caballus* | modern | Debao pony | Guangxi Zhuang Autonomous Region, China | East Asia | 0 |
| 38 | MG001443 | *Equus ferus caballus* | modern | Debao pony | Guangxi Zhuang Autonomous Region, China | East Asia | 0 |
| 39 | MG001423 | *Equus ferus caballus* | modern | Elunchun | The Inner Mongolia Autonomous Region, China | East Asia | 0 |
| 40 | MG001432 | *Equus ferus caballus* | modern | Guizhou | Guizhou Province, China | East Asia | 0 |
| 41 | KT596764 | *Equus ferus caballus* | modern | Hequ | The northwestern Tibetan plateau, China | East Asia | 0 |
| 42 | MG426188 | *Equus ferus caballus* | modern | Hequ | The northwestern Tibetan plateau, China | East Asia | 0 |
| 43 | AY584828 | *Equus ferus caballus* | modern | Jeju | Jeju Island, South Korea | East Asia | 0 |
| 44 | KF038159 | *Equus ferus caballus* | modern | Jeju | Jeju Island, South Korea | East Asia | 0 |
| 45 | KF038160 | *Equus ferus caballus* | modern | Jeju | Jeju Island, South Korea | East Asia | 0 |
| 46 | KF038161 | *Equus ferus caballus* | modern | Jeju | Jeju Island, South Korea | East Asia | 0 |
| 47 | KF038162 | *Equus ferus caballus* | modern | Jeju | Jeju Island, South Korea | East Asia | 0 |
| 48 | KF038163 | *Equus ferus caballus* | modern | Jeju | Jeju Island, South Korea | East Asia | 0 |
| 49 | KF038164 | *Equus ferus caballus* | modern | Jeju | Jeju Island, South Korea | East Asia | 0 |
| 50 | KT998647 | *Equus ferus caballus* | modern | Jianchang | Sichuan  Province, China | East Asia | 0 |
| 51 | MG001428 | *Equus ferus caballus* | modern | Jinjiang | Fujian Province, China | East Asia | 0 |
| 52 | MG001431 | *Equus ferus caballus* | modern | Jinjiang | Fujian Province, China | East Asia | 0 |
| 53 | MG001433 | *Equus ferus caballus* | modern | Jinjiang | Fujian Province, China | East Asia | 0 |
| 54 | HQ439476 | *Equus ferus caballus* | modern | Kustanai | West Kazakhstan | Central Asia | 0 |
| 55 | HQ439474 | *Equus ferus caballus* | modern | Kuznet | Western Siberia, Russia | Northern Asia | 0 |
| 56 | HQ439475 | *Equus ferus caballus* | modern | Kuznet | Western Siberia, Russia | Northern Asia | 0 |
| 57 | MG001413 | *Equus ferus caballus* | modern | Lijiang | Yunnan Province, China | East Asia | 0 |
| 58 | MG001418 | *Equus ferus caballus* | modern | Lijiang | Yunnan Province, China | East Asia | 0 |
| 59 | MG001438 | *Equus ferus caballus* | modern | Lijiang | Yunnan Province, China | East Asia | 0 |
| 60 | MG001437 | *Equus ferus caballus* | modern | Mongolian (Menggu) | The Inner Mongolia Autonomous Region, China | East Asia | 0 |
| 61 | KT368741 | *Equus ferus caballus* | modern | Mongolian | Mongolia | East Asia | 0 |
| 62 | KF038165 | *Equus ferus caballus* | modern | Mongolian | Mongolia | East Asia | 0 |
| 63 | KF038166 | *Equus ferus caballus* | modern | Mongolian | Mongolia | East Asia | 0 |
| 64 | EF597512 | *Equus ferus caballus* | modern | TibetanZhongdian | Zhongdian County, Yunnan province, China | East Asia | 0 |
| 65 | EF597514 | *Equus ferus caballus* | modern | TibetanDeqin | Deqin County, Yunnan province, China | East Asia | 0 |
| 66 | EF597513 | *Equus ferus caballus* | modern | TibetanNaqu | Prefecture-level city Naqu, Tibetan province, China | East Asia | 0 |
| 67 | MF925712 | *Equus ferus caballus* | modern | Sanhe | The Inner Mongolia Autonomous Region, China | East Asia | 0 |
| 68 | MG001415 | *Equus ferus caballus* | modern | Tengchong | Yunnan Province, China | East Asia | 0 |
| 69 | MG001425 | *Equus ferus caballus* | modern | Tengchong | Yunnan Province, China | East Asia | 0 |
| 70 | MG001427 | *Equus ferus caballus* | modern | Tengchong | Yunnan Province, China | East Asia | 0 |
| 71 | MG001446 | *Equus ferus caballus* | modern | Tengchong | Yunnan Province, China | East Asia | 0 |
| 72 | HQ439467 | *Equus ferus caballus* | modern | Yakut | Sakha Republic, Russia | Northern Asia | 0 |
| 73 | KT368727 | *Equus ferus caballus* | modern | Yakut | Sakha Republic, Russia | Northern Asia | 0 |
| 74 | KT368728 | *Equus ferus caballus* | modern | Yakut | Sakha Republic, Russia | Northern Asia | 0 |
| 75 | KT368729 | *Equus ferus caballus* | modern | Yakut | Sakha Republic, Russia | Northern Asia | 0 |
| 76 | KT368730 | *Equus ferus caballus* | modern | Yakut | Sakha Republic, Russia | Northern Asia | 0 |
| 77 | KT368731 | *Equus ferus caballus* | modern | Yakut | Sakha Republic, Russia | Northern Asia | 0 |
| 78 | KT368732 | *Equus ferus caballus* | modern | Yakut | Sakha Republic, Russia | Northern Asia | 0 |
| 79 | KT368733 | *Equus ferus caballus* | modern | Yakut | Sakha Republic, Russia | Northern Asia | 0 |
| 80 | KT368734 | *Equus ferus caballus* | modern | Yakut | Sakha Republic, Russia | Northern Asia | 0 |
| 81 | KT368735 | *Equus ferus caballus* | modern | Yakut | Sakha Republic, Russia | Northern Asia | 0 |
| 82 | KT368736 | *Equus ferus caballus* | modern | Yakut | Sakha Republic, Russia | Northern Asia | 0 |
| 83 | KT368737 | *Equus ferus caballus* | modern | Yakut | Sakha Republic, Russia | Northern Asia | 0 |
| 84 | KT368738 | *Equus ferus caballus* | modern | Yakut | Sakha Republic, Russia | Northern Asia | 0 |
| 85 | MG001421 | *Equus ferus caballus* | modern | Yanqi | Xinjiang Province, China | East Asia | 0 |
| 86 | MG001436 | *Equus ferus caballus* | modern | Yanqi | Xinjiang Province, China | East Asia | 0 |
| 87 | MG001442 | *Equus ferus caballus* | modern | Yanqi | Xinjiang Province, China | East Asia | 0 |
| 88 | MG001420 | *Equus ferus caballus* | modern | Yili | Xinjiang Province, China | East Asia | 0 |
| 89 | MG001429 | *Equus ferus caballus* | modern | Yili | Xinjiang Province, China | East Asia | 0 |
| 90 | MG001434 | *Equus ferus caballus* | modern | Yili | Xinjiang Province, China | East Asia | 0 |
| 91 | MG001414 | *Equus ferus caballus* | modern | Yimen | Yunnan Province, China | East Asia | 0 |
| 92 | MG001441 | *Equus ferus caballus* | modern | Yimen | Yunnan Province, China | East Asia | 0 |
| 93 | MG001448 | *Equus ferus caballus* | modern | Yimen | Yunnan Province, China | East Asia | 0 |
| 94 | MG001419 | *Equus ferus caballus* | modern | Yingjiang | Yunnan Province, China | East Asia | 0 |
| 95 | MG001430 | *Equus ferus caballus* | modern | Yingjiang | Yunnan Province, China | East Asia | 0 |
| 96 | MG001424 | *Equus ferus caballus* | modern | Yunnan pony | Yunnan Province, China | East Asia | 0 |
| 97 | MG001422 | *Equus ferus caballus* | modern | Zhaotong | Yunnan Province, China | East Asia | 0 |
| 98 | MG001435 | *Equus ferus caballus* | modern | Zhaotong | Yunnan Province, China | East Asia | 0 |
| 99 | MG001440 | *Equus ferus caballus* | modern | Zhaotong | Yunnan Province, China | East Asia | 0 |
| 100 | MG001439 | *Equus ferus caballus* | modern | TibetanChangdu | Tibetan province, China | East Asia | 0 |
| 101 | JN398377 1 | *Equus ferus caballus* | modern | Chincoteague Pony | - | North America | 0 |
| 102 | JN398378 2 | *Equus ferus caballus* | modern | Caspian Pony | - | Middle East | 0 |
| 103 | JN398379 3 | *Equus ferus caballus* | modern | Maremmano | - | Southern Europe | 0 |
| 104 | JN398380 4 | *Equus ferus caballus* | modern | Arabian | - | Middle East | 0 |
| 105 | JN398381 5 | *Equus ferus caballus* | modern | Maremmano | - | Southern Europe | 0 |
| 106 | JN398382 6 | *Equus ferus caballus* | modern | Maremmano | - | Southern Europe | 0 |
| 107 | JN398383 7 | *Equus ferus caballus* | modern | Unspecified Iranian Breed | - | Middle East | 0 |
| 108 | JN398384 8 | *Equus ferus caballus* | modern | Unspecified Syrian Breed | - | Middle East | 0 |
| 109 | JN398385 9 | *Equus ferus caballus* | modern | Akhal-Teke | - | Central Asia | 0 |
| 110 | JN398386 10 | *Equus ferus caballus* | modern | Westphalian | - | Central Europe | 0 |
| 111 | JN398387 11 | *Equus ferus caballus* | modern | Maremmano | - | Southern Europe | 0 |
| 112 | JN398388 12 | *Equus ferus caballus* | modern | Maremmano | - | Southern Europe | 0 |
| 113 | JN398389 13 | *Equus ferus caballus* | modern | Unspecified Syrian Breed | - | Middle East | 0 |
| 114 | JN398390 14 | *Equus ferus caballus* | modern | Unspecified Italian Breed | - | Southern Europe | 0 |
| 115 | JN398391 15 | *Equus ferus caballus* | modern | Unspecified Syrian Breed | - | Middle East | 0 |
| 116 | JN398392 16 | *Equus ferus caballus* | modern | Arabian | - | Middle East | 0 |
| 117 | JN398393 17 | *Equus ferus caballus* | modern | Akhal-Teke | - | Central Asia | 0 |
| 118 | JN398394 18 | *Equus ferus caballus* | modern | Unspecified Syrian Breed | - | Middle East | 0 |
| 119 | JN398395 19 | *Equus ferus caballus* | modern | Unspecified Iranian Breed | - | Middle East | 0 |
| 120 | JN398396 20 | *Equus ferus caballus* | modern | Suffolk Punch | - | Northern Europe | 0 |
| 121 | JN398397 21 | *Equus ferus caballus* | modern | Maremmano | - | Southern Europe | 0 |
| 122 | JN398398 22 | *Equus ferus caballus* | modern | Norwegian Fjord | - | Northern Europe | 0 |
| 123 | JN398399 23 | *Equus ferus caballus* | modern | Icelandic | - | Northern Europe | 0 |
| 124 | JN398400 24 | *Equus ferus caballus* | modern | Icelandic | - | Northern Europe | 0 |
| 125 | JN398401 25 | *Equus ferus caballus* | modern | Maremmano | - | Southern Europe | 0 |
| 126 | JN398404 28 | *Equus ferus caballus* | modern | Akhal-Teke | - | Central Asia | 0 |
| 127 | JN398405 30 | *Equus ferus caballus* | modern | Unspecified Iranian Breed | - | Middle East | 0 |
| 128 | JN398406 31 | *Equus ferus caballus* | modern | Arabian | - | Middle East | 0 |
| 129 | JN398407 32 | *Equus ferus caballus* | modern | Giara | Sardinia island, Italy | Southern Europe | 0 |
| 130 | JN398408 33 | *Equus ferus caballus* | modern | Unspecified Syrian Breed | - | Middle East | 0 |
| 131 | JN398409 34 | *Equus ferus caballus* | modern | Unspecified Italian Breed | - | Southern Europe | 0 |
| 132 | JN398410 35 | *Equus ferus caballus* | modern | Akhal-Teke | - | Central Asia | 0 |
| 133 | JN398411 36 | *Equus ferus caballus* | modern | Giara | Sardinia island, Italy | Southern Europe | 0 |
| 134 | JN398412 37 | *Equus ferus caballus* | modern | Arabian | - | Middle East | 0 |
| 135 | JN398413 38 | *Equus ferus caballus* | modern | Maremmano | - | Southern Europe | 0 |
| 136 | JN398414 39 | *Equus ferus caballus* | modern | Unspecified Iranian Breed | - | Middle East | 0 |
| 137 | JN398415 40 | *Equus ferus caballus* | modern | Unspecified Iranian Breed | - | Middle East | 0 |
| 138 | JN398416 41 | *Equus ferus caballus* | modern | Caspian Pony | - | Middle East | 0 |
| 139 | JN398417 42 | *Equus ferus caballus* | modern | Trakhener | - | Northern Europe | 0 |
| 140 | JN398418 43 | *Equus ferus caballus* | modern | Maremmano | - | Southern Europe | 0 |
| 141 | JN398419 44 | *Equus ferus caballus* | modern | Unspecified Iranian Breed | - | Middle East | 0 |
| 142 | JN398420 45 | *Equus ferus caballus* | modern | Belgian Draft | - | Central Europe | 0 |
| 143 | JN398421 48 | *Equus ferus caballus* | modern | American Paint Horse | - | North America | 0 |
| 144 | JN398422 46 | *Equus ferus caballus* | modern | Akhal-Teke | - | Central Asia | 0 |
| 145 | JN398423 47 | *Equus ferus caballus* | modern | Unspecified Iranian Breed | - | Middle East | 0 |
| 146 | JN398424 49 | *Equus ferus caballus* | modern | Akhal-Teke | - | Central Asia | 0 |
| 147 | JN398425 50 | *Equus ferus caballus* | modern | Unspecified Italian Breed | - | Southern Europe | 0 |
| 148 | JN398427 51 | *Equus ferus caballus* | modern | Maremmano | - | Southern Europe | 0 |
| 149 | JN398426 52 | *Equus ferus caballus* | modern | Maremmano | - | Southern Europe | 0 |
| 150 | JN398428 53 | *Equus ferus caballus* | modern | Caspian Pony | - | Middle East | 0 |
| 151 | JN398429 54 | *Equus ferus caballus* | modern | Oldenburg | - | Northern Europe | 0 |
| 152 | JN398430 55 | *Equus ferus caballus* | modern | Andalusian | - | Southern Europe | 0 |
| 153 | JN398431 56 | *Equus ferus caballus* | modern | Silesian | - | Central Europe | 0 |
| 154 | JN398432 57 | *Equus ferus caballus* | modern | Maremmano | - | Southern Europe | 0 |
| 155 | JN398433 58 | *Equus ferus caballus* | modern | Unspecified Iranian Breed | - | Middle East | 0 |
| 156 | JN398434 59 | *Equus ferus caballus* | modern | Arabian | - | Middle East | 0 |
| 157 | JN398435 60 | *Equus ferus caballus* | modern | Akhal-Teke | - | Central Asia | 0 |
| 158 | JN398436 61 | *Equus ferus caballus* | modern | Caspian Pony | - | Middle East | 0 |
| 159 | JN398437 63 | *Equus ferus caballus* | modern | Maremmano | - | Southern Europe | 0 |
| 160 | JN398438 64 | *Equus ferus caballus* | modern | Friesian | - | Central Europe | 0 |
| 161 | JN398439 62 | *Equus ferus caballus* | modern | Clydesdale | - | Northern Europe | 0 |
| 162 | JN398440 65 | *Equus ferus caballus* | modern | English Shire | - | Northern Europe | 0 |
| 163 | JN398441 66 | *Equus ferus caballus* | modern | Saddlebred | - | North America | 0 |
| 164 | JN398442 67 | *Equus ferus caballus* | modern | Exmoor Pony | - | Northern Europe | 0 |
| 165 | JN398443 68 | *Equus ferus caballus* | modern | Andalusian | - | Southern Europe | 0 |
| 166 | JN398444 69 | *Equus ferus caballus* | modern | Unspecified Iranian Breed | - | Middle East | 0 |
| 167 | JN398445 70 | *Equus ferus caballus* | modern | Unspecified Iranian Breed | - | Middle East | 0 |
| 168 | JN398446 71 | *Equus ferus caballus* | modern | Unspecified Iranian Breed | - | Middle East | 0 |
| 169 | JN398447 72 | *Equus ferus caballus* | modern | Caspian Pony | - | Middle East | 0 |
| 170 | JN398448 73 | *Equus ferus caballus* | modern | Arabian | - | Middle East | 0 |
| 171 | JN398449 74 | *Equus ferus caballus* | modern | Akhal-Teke | - | Central Asia | 0 |
| 172 | JN398450 75 | *Equus ferus caballus* | modern | Akhal-Teke | - | Central Asia | 0 |
| 173 | JN398451 76 | *Equus ferus caballus* | modern | Unspecified Iranian Breed | - | Middle East | 0 |
| 174 | JN398452 77 | *Equus ferus caballus* | modern | Akhal-Teke | - | Central Asia | 0 |
| 175 | JN398453 78 | *Equus ferus caballus* | modern | Akhal-Teke | - | Central Asia | 0 |
| 176 | JN398454 80 | *Equus ferus caballus* | modern | Maremmano | - | Southern Europe | 0 |
| 177 | JN398455 81 | *Equus ferus caballus* | modern | Unspecified Iranian Breed | - | Middle East | 0 |
| 178 | JN398456 82 | *Equus ferus caballus* | modern | Maremmano | - | Southern Europe | 0 |
| 179 | JN398457 83 | *Equus ferus caballus* | modern | Unspecified Iranian Breed | - | Middle East | 0 |
| 180 | AP012268 | *Equus ferus przewalskii* | modern | studbook number 668, maternal lineage Bijsk/2 | ZSL Whipsnade Zoo, Bedfordshire county, England | Western Europe | 0 |
| 181 | AP012267 | *Equus ferus przewalskii* | modern | studbook number 319, maternal lineage Staraja II | Catskill Game Farm, New York state, USA | North America | 0 |
| 182 | KT368742 | *Equus ferus przewalskii* | modern | studbook number 524 | Askania-Nova biosphere reserve, Kherson Oblast, Ukraine | Eastern Europe | 0 |
| 183 | KT368743 | *Equus ferus przewalskii* | modern | studbook number 528 | Askania-Nova biosphere reserve, Kherson Oblast, Ukraine | Eastern Europe | 0 |
| 184 | KT368744 | *Equus ferus przewalskii* | modern | studbook number 285, maternal lineage Orlica III | Askania-Nova biosphere reserve, Kherson Oblast, Ukraine | Eastern Europe | 0 |
| 185 | KT368745 | *Equus ferus przewalskii* | modern | studbook number 966 | Kosov farm, Ivano-Frankivsk Oblast, Ukraine | Eastern Europe | 0 |
| 186 | KT368746 | *Equus ferus przewalskii* | modern | studbook number 281 | Paris, France | Western Europe | 0 |
| 187 | KT368747 | *Equus ferus przewalskii* | modern | studbook number 615 | ZSL Whipsnade Zoo, Bedfordshire county, England | Western Europe | 0 |
| 188 | KT368748 | *Equus ferus przewalskii* | modern | studbook number 293 | Catskill Game Farm, New York state, USA | North America | 0 |
| 189 | KT368749 | *Equus ferus przewalskii* | modern | studbook number 339 | Catskill Game Farm, New York state, USA | North America | 0 |
| 190 | KT368750 | *Equus ferus przewalskii* | modern | studbook number 533 | Catskill Game Farm, New York state, USA | North America | 0 |
| 191 | KT368751 | *Equus ferus przewalskii* | modern | studbook number 159 | Munich, the Free State of Bavaria, Germany | Central Europe | 0 |
| 192 | KT368752 | *Equus ferus przewalskii* | modern | studbook number 274 | Copenhagen, Denmark | Northern Europe | 0 |
| 193 | KT368757 | *Equus ferus przewalskii* | modern | Przewalski x Domesticated F1 hybrid | San Diego Zoo’s Institute for Conservation Research, the County of San Diego, the State of California, the West Pacific States, USA | North America | 0 |
| 194 | HQ439484 | *Equus ferus przewalskii* | modern | - | - | East Asia | 0 |
| 195 | JN398402 | *Equus ferus przewalskii* | modern | - | - | Central Asia | 0 |
| 196 | JN398403 | *Equus ferus przewalskii* | modern | - | - | Central Asia | 0 |
| 197 | KT757761 | *Equus ferus przewalskii* | modern | - | The Natural History Museum of Denmark, the Capital Region of Denmark, the Kingdom of Denmark | Northern Europe | 0 |
| 198 | NC_024030 | *Equus ferus przewalskii* | modern | - | Tokyo University of Agriculture, Hokkaidō region, Japan | East Asia | 0 |
| 199 | AP013095 | *Equus ferus przewalskii* | modern | - | Tokyo University of Agriculture, Hokkaidō region, Japan | East Asia | 0 |
| 200 | NC_001788 | *Equus asinus asinus* | modern | - | - | Northern Europe | 0 |
