# Supplementary figures and images for: Traces of Late Bronze and Early Iron Age Mongolian Horse Mitochondrial Lineages in Modern Populations
Source: Genes (Basel). 2021 Mar 12;12(3):412. doi: 10.3390/genes12030412 (PMC8000342; doi:10.3390/genes12030412)

95% HPD (height posterior density)

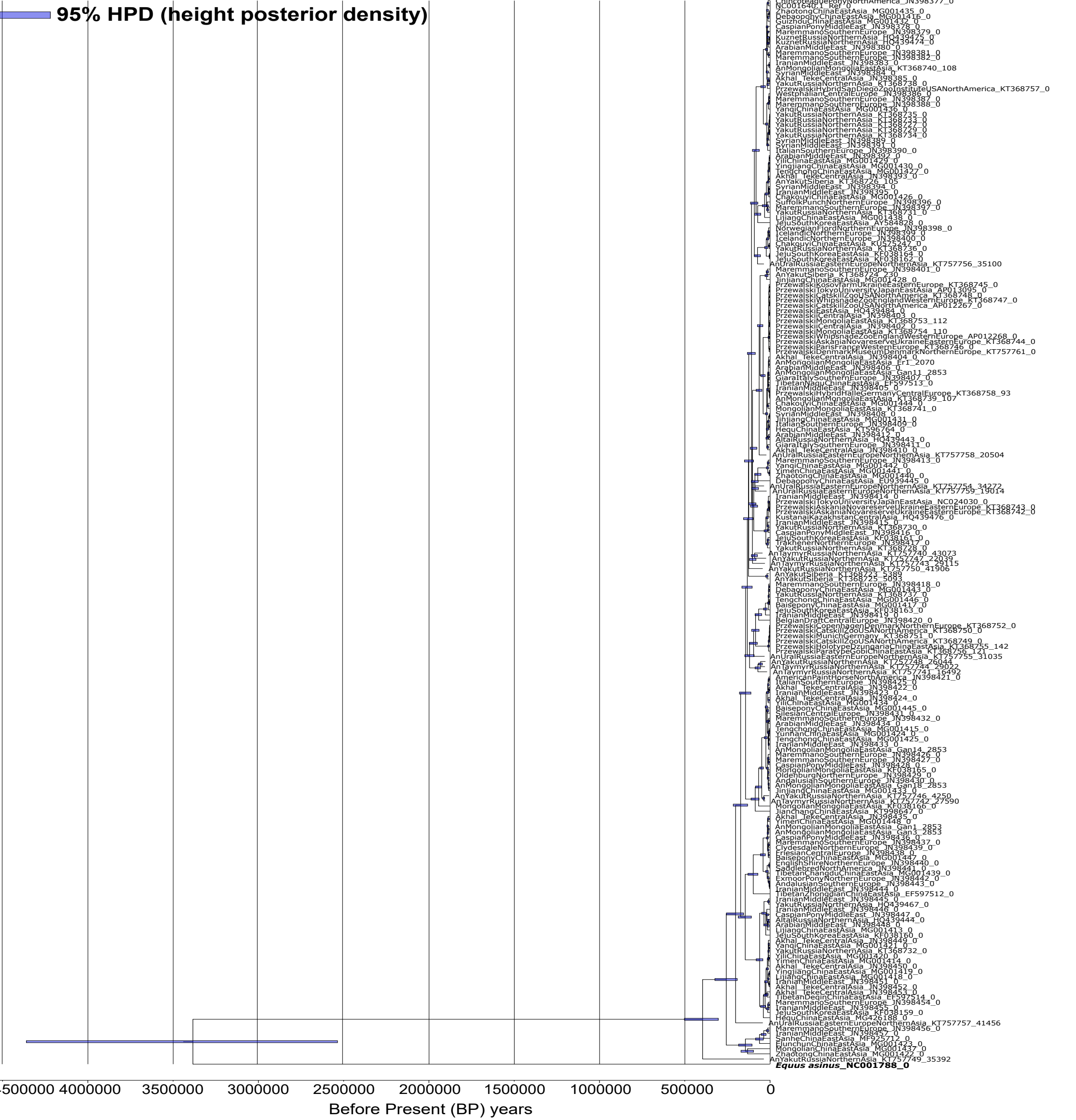

Supplement: Supplementary file 1 [file genes-12-00412-s001.zip › Figure S4. Initial phylogenetic tree (with Equus asinus) built here showing the 95% height posterior density of age estimates..pdf]

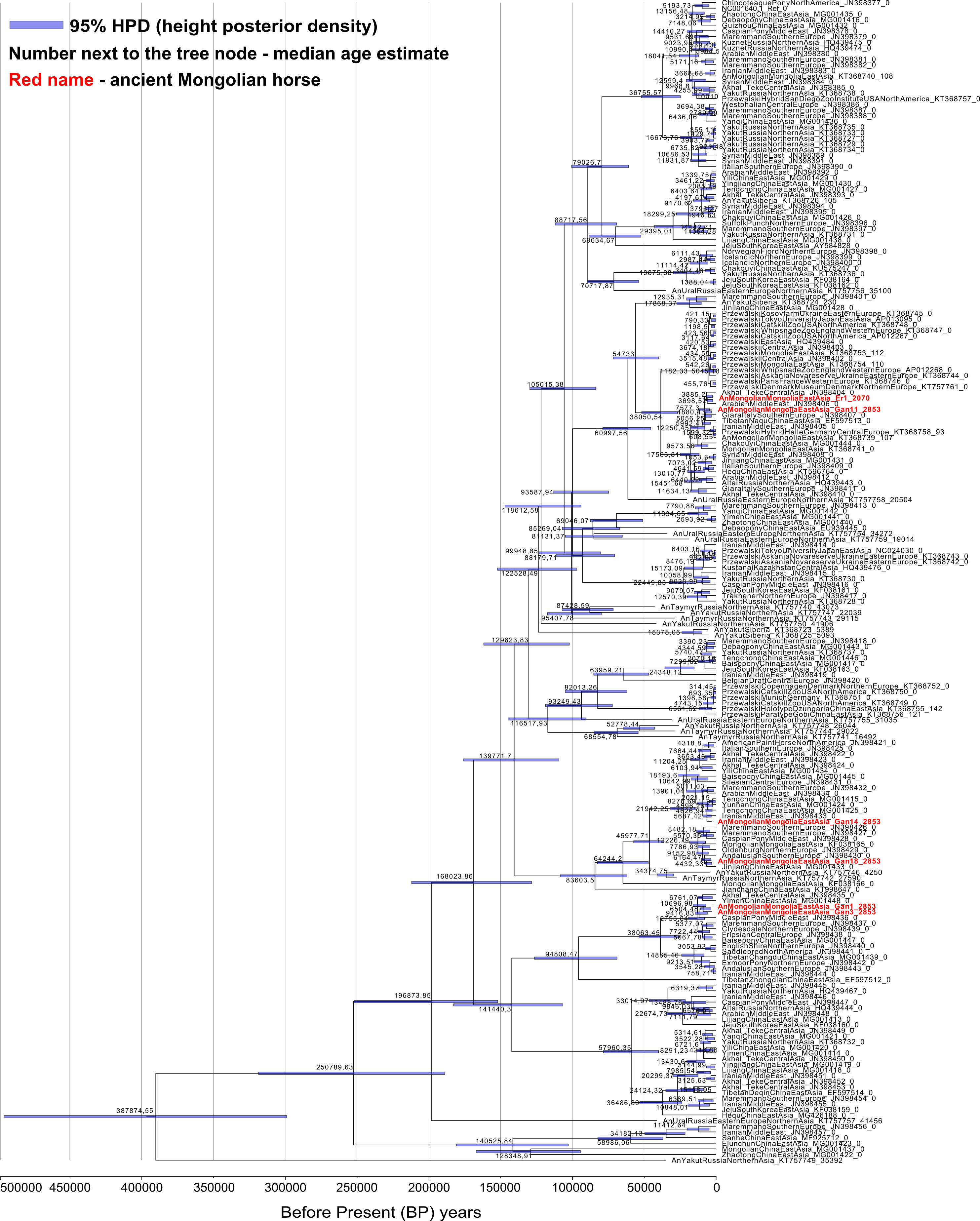

Supplement: Supplementary file 1 [file genes-12-00412-s001.zip › Figure S5. Initial phylogenetic tree (without Equus asinus) built here showing the 95% height posterior density of age estimates..png]
